# Unveiling wheat growth promotion potential of phosphate solubilizing *Pantoea agglomerans* PS1 and PS2 through genomic, physiological, and metagenomic characterizations

**DOI:** 10.3389/fmicb.2024.1467082

**Published:** 2024-09-09

**Authors:** Pinki Sharma, Rajesh Pandey, Nar Singh Chauhan

**Affiliations:** ^1^Department of Biochemistry, Maharshi Dayanand University, Rohtak, India; ^2^INtegrative GENomics of HOst-PathogEn (INGEN-HOPE) Laboratory, CSIR-Institute of Genomics and Integrative Biology (CSIR-IGIB), Delhi, India; ^3^Academy of Scientific and Innovative Research (AcSIR), Ghaziabad, India

**Keywords:** wheat rhizosphere, phosphate solubilizing bacteria, biofertilizers, comparative genomics, rhizosphere microbiota, microbiota engineering, sustainable agriculture

## Abstract

**Introduction:**

Phosphorus is an abundant element in the earth’s crust and is generally found as complex insoluble conjugates. Plants cannot assimilate insoluble phosphorus and require external supplementation as chemical fertilizers to achieve a good yield. Continuous use of fertilizers has impacted soil ecology, and a sustainable solution is needed to meet plant elemental requirements. Phosphate solubilizing microbes could enhance phosphorus bioavailability for better crop production and can be employed to attain sustainable agriculture practices.

**Methods:**

The current study unveils the biofertilizer potential of wheat rhizospheric bacteria through physiological, taxonomic, genomic, and microbiomics experimentations.

**Results and Discussion:**

Culture-dependent exploration identified phosphate-solubilizing PS1 and PS2 strains from the wheat rhizosphere. These isolates were rod-shaped, gram-negative, facultative anaerobic bacteria, having optimum growth at 37°C and pH 7. Phylogenetic and phylogenomic characterization revealed their taxonomic affiliation as *Pantoea agglomerans* subspecies PS1 & PS2. Both isolates exhibited good tolerance against saline (>10% NaCl (w/v), >11.0% KCl (w/v), and >6.0% LiCl (w/v)), oxidizing (>5.9% H_2_O_2_ (v/v)) conditions. PS1 and PS2 genomes harbor gene clusters for biofertilization features, root colonization, and stress tolerance. PS1 and PS2 showed nitrate reduction, phosphate solubilization, auxin production, and carbohydrate utilization properties. Treatment of seeds with PS1 and PS2 significantly enhanced seed germination percentage (*p* = 0.028 and *p* = 0.008, respectively), number of tillers (*p* = 0.0018), number of leaves (*p* = 0.0001), number of spikes (*p* = 0.0001) and grain production (*p* = 0.0001). Wheat rhizosphere microbiota characterizations indicated stable colonization of PS1 and PS2 strains in treated seeds at different feek stages. Pretreatment of seeds with both strains engineered the wheat rhizosphere microbiota by recruiting plant growth-promoting microbial groups. *In vitro*, *In vivo*, and microbiota characterization studies indicated the biofertilizer potential of *Pantoea* sp. PS1 & PS2 to enhance wheat crop production. The employment of these strains could fulfill plant nutrient requirements and be a substitute for chemical fertilizers for sustainable agriculture.

## Introduction

Phosphorus, one of the most essential macronutrients after nitrogen, plays a vital role in the growth and development of plants. Phosphorus is abundantly available in both inorganic and organic forms in soil (Sharma et al., 2013). However, phosphorous is generally found in bounded forms, making it difficult for vascular uptake (Rengel and Marschner, 2005). Chemical fertilizers seem to be an alternative approach to resolving the issue. However, plants can utilize only 5–10% phosphate supplied as fertilizer (Omo-Okoro et al., 2023). Highly reactive phosphate anions in chemical fertilizers interact with Ca^2+^, Al^3+^, and Fe^3+^ in soil, resulting in insoluble phosphate salt complexes (Schnug and Haneklaus, 2016). Continuous usage of phosphorous fertilizers to meet plant growth requirements increases the abundance of phosphate complexes in the soil, which results in phosphate-induced zinc (Cakmak and Marschner, 1987) and iron deficiency (Hue and Nakamura, 1988) and related consequences. Therefore, it is an obligation to maintain a balanced level of phosphorus in soil instead of blindly adding chemical fertilizers as phosphorus supplements. It could be achieved by enhancing the bioavailability of soil phosphates. Efforts are being made to develop novel chemical formulations (Duarah et al., 2011), nano-fertilizers (Basavegowda and Baek, 2021), and stimulating plant metabolic machinery (Pang et al., 2024) for improved vascular uptake. Despite these breakthroughs, their mass-scale employment in agriculture practices is challenged due to high cost, scalability, and environmental concerns (Boix-Fayos and De Vente, 2023). Researchers are exploring alternative sustainable solutions to overcome the nutrient bioavailability challenges (Overcoming barriers to sustainable, healthy diets, 2022). Phosphate-solubilizing bacteria (PSB) hold great promise to overcome this issue.

The plant rhizosphere harbors diversified microbes important for plant growth, development, and stress management (Sharma et al., 2021). Culture-based studies have isolated phosphate-solubilizing bacteria and investigated their importance in the solubilization of chemically bound phosphates (Alori et al., 2017; Chen and Liu, 2019). Various bacterial members belonging to *Azotobacter, Agrobacterium, Arthrobacter, Bradyrhizobium, Burkholderia, Alcaligenes, Bacillus, Clromobacterium, Flavobacterium, Micrococcus, Enterobacter, Pantoea*, and *Ochrobactrum* genus were characterized for phosphate solubilizing properties (Cheng et al., 2023). However, their efficacy in meeting the plant nutrient requirement, stability under saline and oxidative stress, interaction with different host plants during plant growth stages, and influence on plant’s native microbiome are yet to be addressed before their potential application for sustainable agriculture (Rasul et al., 2021). Genetic and physiological characterizations of *Pantoea* sp. confirmed wheat growth promotion potential (Rasul et al., 2021) and successful colonization (Remus et al., 2000). However, their influence on wheat rhizosphere microbiota still awaits exploration. Plant rhizosphere microbiota plays a vital role in plant growth and development (Macik et al., 2020). Metagenomic elucidation of wheat rhizosphere microbiota would identify key microbial partners and factors governing microbial community dynamics (Macik et al., 2020; Kong and Liu, 2022). This knowledge is pivotal for developing targeted techniques for enhancing agricultural productivity and advancing bioremediation strategies (Kong and Liu, 2022). An in-depth characterization of potential plant growth-promoting bacteria is essential for proper risk assessment before employment in sustainable agriculture. Hereby, the current study explored the wheat rhizosphere microbiome for phosphate solubilizing microbes and the impact of these microbes on wheat growth before employing them for sustainable agriculture.

## Materials and methods

### Isolation and screening of phosphate solubilizing wheat rhizosphere bacteria

Rhizospheric soil samples were collected from wheat plants cultivated in an experimental field in the botanical garden (28° 52′ 44” NL and 76° 37′ 19″ EL) at Maharshi Dayanand University, Rohtak, Haryana, India. Wheat rhizospheric bacteria were isolated following previously standardized conditions (Sharma et al., 2024). The phosphate solubilizing potential of wheat rhizosphere bacteria was screened as per the Pikovskaya agar plate screening (Pikovskaya, 1948).

### Taxonomic, physiological, and biochemical characterization of phosphate solubilizing bacteria

The alkali lysis method (Chauhan et al., 2009) was employed for DNA isolation of PSB. Qualitative and quantitative analyses of the DNA were performed using agarose gel electrophoresis and Qubit HS DNA estimation kits (Invitrogen, USA), respectively. The 16S rRNA gene was amplified and sequenced to determine the taxonomic affiliation of the microbes following standardized protocols (Yadav et al., 2023). The Gram staining kit (K001-1KT, Himedia) was used to characterize phosphate solubilizing bacteria (PSB). Optimal growth conditions of PSB were examined across various pH (3, 4, 5, 7, 8, 9, 10, 11 and 12) and temperature ranges (10°C, 15°C, 20°C, 25°C, 30°C, 35°C, 40°C, 45°C, 50°C, 55°C, 60°C) (Rasul et al., 2021). The bacterial growth pattern was monitored in LB broth for 48 h at 37°C with continuous shaking at 200 rpm to assess their doubling time (Wang et al., 2015). The substrate utilization preferences of the identified microbes were evaluated using the Hi Carbo kit (Himedia, KB009A-1KT, KB009B-1KT, and KB009C-1KT). Furthermore, the biochemical properties of the strains were assessed through activities for amylase (Swain et al., 2006), catalase (Iwase et al., 2013), pectinase (Oumer and Abate, 2018), cellulase (Kasana et al., 2008), esterase (Ramnath et al., 2017), and protease (Vijayaraghavan et al., 2017). The antibiotic susceptibility profiles of the microbial isolates were determined using the Combi IV kit (Himedia, OD023) and G-VI-plus (Himedia, OD034). Stress response physiology was assessed by subjecting them to salt, arsenic, and oxidative stress (Yadav et al., 2023). Salt stress tolerance was checked after assessing the growth of PS1 and PS2 in LB broth (5 mL) supplemented with different concentrations of NaCl, KCl, and LiCl (Yadav et al., 2023). Oxidative stress tolerance of PS1 and PS2 was assessed after observing their growth in LB broth (5 mL) supplemented with different concentrations of H_2_O_2_ (0, 1.0 mM, 2.5 mM, 5.0 mM, 7.5 mM, 10 mM, 12.5 mM. 15.0 mM, 20.0 mM, and 25 mM) (Sharma et al., 2024). Arsenic stress tolerance of PSB was checked after observing their growth in LB broth (5 mL) supplemented with different concentrations of sodium arsenite and sodium arsenate (Sharma et al., 2024).

### Genome characterisation of *Pantoea agglomerans* PS1 and PS2

PS1 and PS2 bacterial genomes were sequenced using Illumina MiSeq with the Nextera XT DNA Library Prep kit. Sequence curation, genome assembly, basement of genome completeness, genome annotation, and genome map creation were performed as described previously (Rasul et al., 2021; Sharma et al., 2024). CRISPR/Cas in the genome was identified using a CRISPR identifier, and antibiotic-resistance genes were identified using the CARD identifier. Protein features responsible for phosphate solubilization, antibiotic resistance, metal/metalloid resistance, and oxidative stress resistance were identified using the Rapid Annotation using Subsystem Technology (RAST) server.^1^ Pathogenesis potential was assessed using Island Viewer 4 with default parameters. Phylogenomic characterization of PS1 and PS2 with other *Pantoea* sp. was plotted using roary_plots.py v0.1.0.^2^ The core multiple sequence alignment was used to infer the phylogenomic tree using FastTree v2.1.10 (Price et al., 2010).

### Assessment of phosphate solubilizing activity of *Pantoea agglomerans* PS1 and PS2

The phosphate solubilizing capacity of the bacteria was evaluated using the previously described protocol (Behera et al., 2017). The bacterial strains were cultured overnight in the National Botanical Research Institute’s phosphate growth medium (NBRIP) at 37°C with continuous shaking at 200 rpm to assess alkaline phosphatase and acid phosphatase activities.

### The plant growth promotion potential of *Pantoea agglomerans* PS1 and PS2

*P. agglomerans* PS1 and PS2 were screened for nitrate reductase activity (Kim and Seo, 2018), Indole-3-acetic acid (IAA) production (Ehmann, 1977), ammonia production (Bhattacharyya et al., 2020), and siderophore biosynthesis (Himpsl and Mobley, 2019) for the assessment of their bio-fertilization potential.

### Assessment of drought and oxidative stress tolerance ability of microbial isolates

Drought stress tolerance of PS1 and PS2 was assessed as described previously (Elizabeth Mustamu et al., 2023). PS1 and PS2 were cultured at 37°C for 24 h with continuous shaking at 200 rpm in the nutrient broth (NB). A 100ul of microbial culture was inoculated in LB broth (2x) supplemented with a different concentration of Polyethylene glycol (PEG) (0, 5, 10, 20, 30, and 40% (w/v)). The total reaction volume was adjusted to 5 mL by adding sterile double distilled water. Absorbance was read at 600 nm after an incubation of 24 h at 37°C with continuous shaking at 200 rpm. ACC deaminase production activity of microbial isolates was checked with a standardized methodology (Maheshwari et al., 2020) to evaluate their salt-induced oxidative stress tolerance. In brief, microbial cells were initially induced in a 5 mL minimal media (Dworkin and Foster, 1958) followed by harvesting the cell by centrifugation at 16,000 x g for 5 min. Cells were washed with 0.1 M Tris–HCl (pH 7.6) and resuspended in 600 μL of 0.1 M Tris–HCl (pH 8.5). 30 μL of toluene was added to disrupt the cells, followed by vortexing for 30 s. 200 μL of the toluenized cell suspensions were mixed with 20 μL of 0.5 M ACC and incubated at 30°C for 15 min. One mL of 0.56 N HCl was added after the incubation. The mixture was vortexed to remove cell debris by centrifugation at 16,000 rpm for 5 min. One mL of the culture supernatant was combined with 800 μL of HCl (0.56 N) and freshly prepared 300 μL of 2,4-dinitrophenyl hydrazine (DNPH) reagent (0.1 g DNPH in 100 mL of 2 N HCl). The reaction mixture was incubated at 30°C for 30 min followed by the addition of 2 mL of NaOH (2 N). The absorbance was measured at 540 nm. Enzyme activity was calculated using the α-Ketoglutarate standard curve (R = 0.9998).

### Influence of *Pantoea agglomerans* PS1 and PS2 on seed germination under salt stress conditions

The wheat seed germination assays were performed in the presence of *P. agglomerans* PS1 and PS2. Seed pretreatment with bacterial agents to assess their protective effect was performed as described previously (Sharma et al., 2024). Seeds were soaked in overnight-grown bacterial cultures (density of 10^11^ cells/ml) supplemented with varying concentrations of NaCl ranging from 0 to 1.0 M and incubated at 37°C for 16 h. The control seeds were soaked directly at different concentrations of NaCl ranging from 0 to 1.0 M for 16 h at 37°C. Subsequently, the seeds were wrapped in germination sheets and placed in 50 mL culture tubes containing 5 mL Hoagland solution to calculate seed germination percentage (Sharma et al., 2024). Tubes were incubated for 7 days in the dark at room temperature. Seed germination percentage, alpha-amylase activity, root length, and shoot lengths were measured after incubation (Singh and Kayastha, 2014).

### Wheat rhizosphere microbiota profiling

Wheat cultivar 306 seeds treated with *P. agglomerans* PS1 and PS2 were cultivated in an experimental field in the botanical garden at Maharshi Dayanand University, Rohtak (28° 52′ 44” NL and 76° 37′ 19″ EL), Haryana, India. Wheat Growth and development stages are categorized into various Feeks stages.^3^ Wheat roots were harvested at different feeks stages (1.0, 2.0, 3.0, 6.0, 9.0, and 10.5) to assess the impact of *P. agglomerans* PS1 and PS2 across plant growth stages. (Here: Feeks 1.0 is the seedling emergence stage, Feeks 3.0 is the plant tillering stage, Feeks 6.0 is the first node appearance stage, Feeks 9.0 is flag leaf is the visible stage, while Feeks 10.5 is the wheat growth stage when heading complete). Metagenomic DNA of wheat rhizosphere was extracted using the alkaline lysis methodology (Kumar et al., 2016). Metagenomic DNA was purified using a soil DNA purification kit (Himedia, HiPurA). The 16S rRNA gene sequences were amplified from wheat rhizosphere metagenomic DNA using universal primers (27F 5’-AGAGTTTGATCCTGGCTCAG-3′; 1492R 5’-GGTTACCT TGTTACGACTT-3′) (James, 2010). Sequencing of 16S rRNA gene amplicons was performed on nanopore minion MK1C following midnight protocol. Wheat rhizospheric 16S rRNA sequence data was analyzed using Commander 2.0^4^ following the 16S rRNA sequence analysis pipeline.

### Plant growth promoting potential of *Pantoea agglomerans* PS1 and PS2 in experimental field conditions

Seeds were soaked with 5 mL of overnight-grown bacterial cultures (density of 10^11^ cells/ml). Preincoucalted seeds were planted in the experimental soil bed and allowed to grow till harvest stages. Wheat cultivar WC-306 plant roots treated with *P. agglomerans* PS1 *and* PS2 were harvested at different feeks and assessed for phosphate solubilizing ability (Behera et al., 2017), nitrate reductase activity (Kim and Seo, 2018), total sugar content (Ludwig and Goldberg, 1956) and reducing sugar content (Khatri and Chhetri, 2020) in reference to non-treated WC-306 plant roots. Additionally, the number of tillers formed, the number of leaves per plant, the number of spikes per plant, spike length, the number of spikelets per spike, and grain yield were also estimated in both treated and non-treated WC-306 plants to assess the impact of *Pantoea agglomerans* strains on plant growth and yield.

### Statistical analysis

All experiments were carried out in replicates. SIGMA plot 15 was employed to perform statistical evaluations and for graphical representations of the datasets. ANOVA analysis was performed to calculate significance among microbial treated and non-treated groups using Systat Software SigmaPlot 15.

## Results

### Isolation and screening of phosphate solubilizing wheat rhizosphere bacteria

The wheat rhizospheric soil had a pH of 7.3 ± 0.15275, a temperature of 22.6°C ± 0.50332, and a moisture content of 11.5% ± 1.10014 (w/w). Twelve morphologically distinct bacteria were isolated from the wheat rhizosphere. Plate screening assay for phosphate solubilization property showed that only two isolates, PS1 and PS2 led to the development of halo-zones of 23 ± 0.327 mm and 25 ± 0.47735 mm, respectively.

### Taxonomic, physiological, and biochemical characterization of phosphate solubilizing bacteria

**Figure 1:**
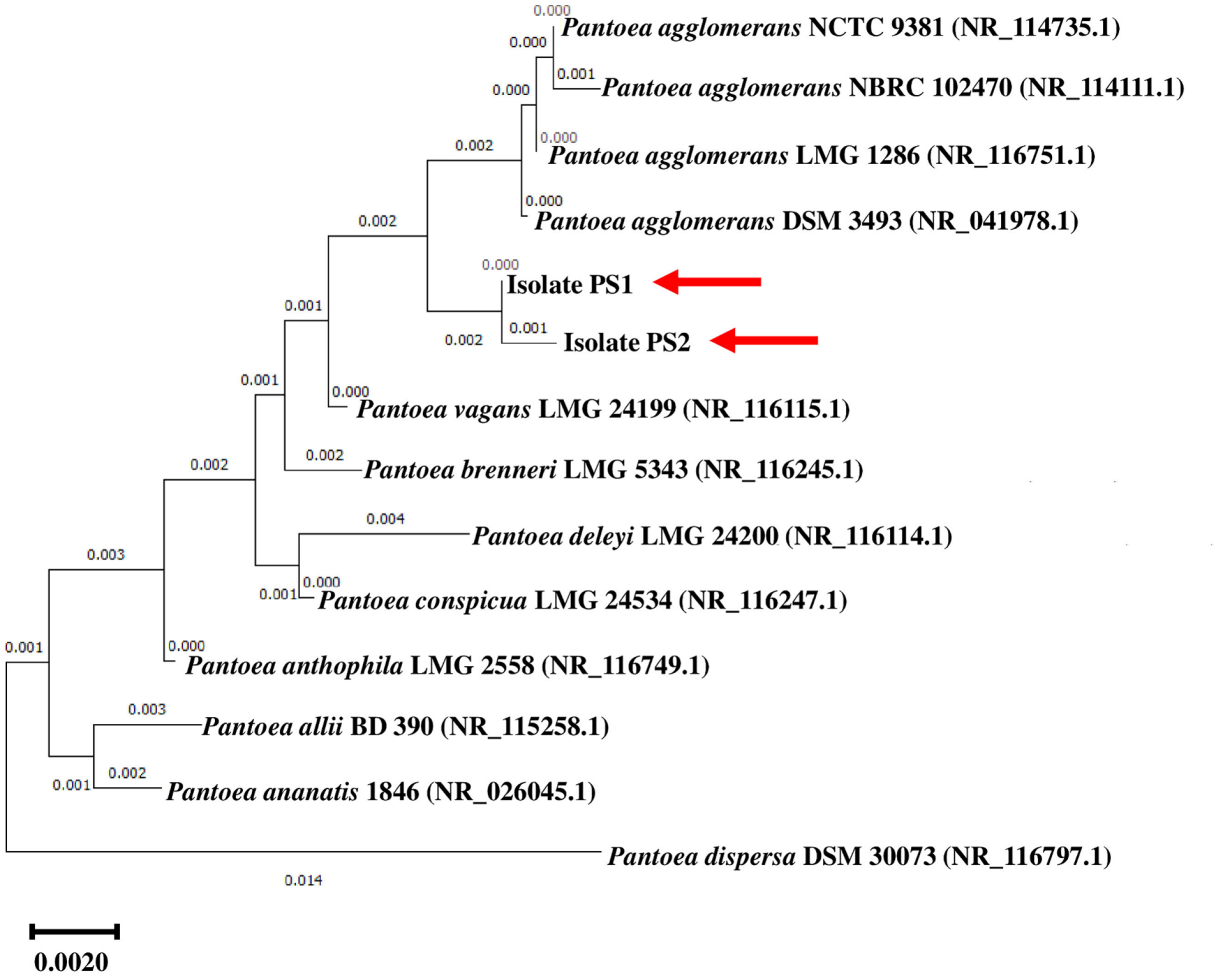
Phylogenetic affiliation of microbial isolates *Pantoea agglomerans* PS1 *and* PS2 with the other *Pantoea* species. The phylogenetic tree was constructed with the neighbor-joining method of phylogenetics using 1,000 bootstrap replications of the 16S rRNA gene sequences of *Pantoea agglomerans* PS1 *and Pantoea agglomerans* PS2 and NCBI database homologs using MEGA-X software. Out-group was represented by *Pantoea ananatis* 1846 SSU rRNA gene sequence.

**Figure 2:**
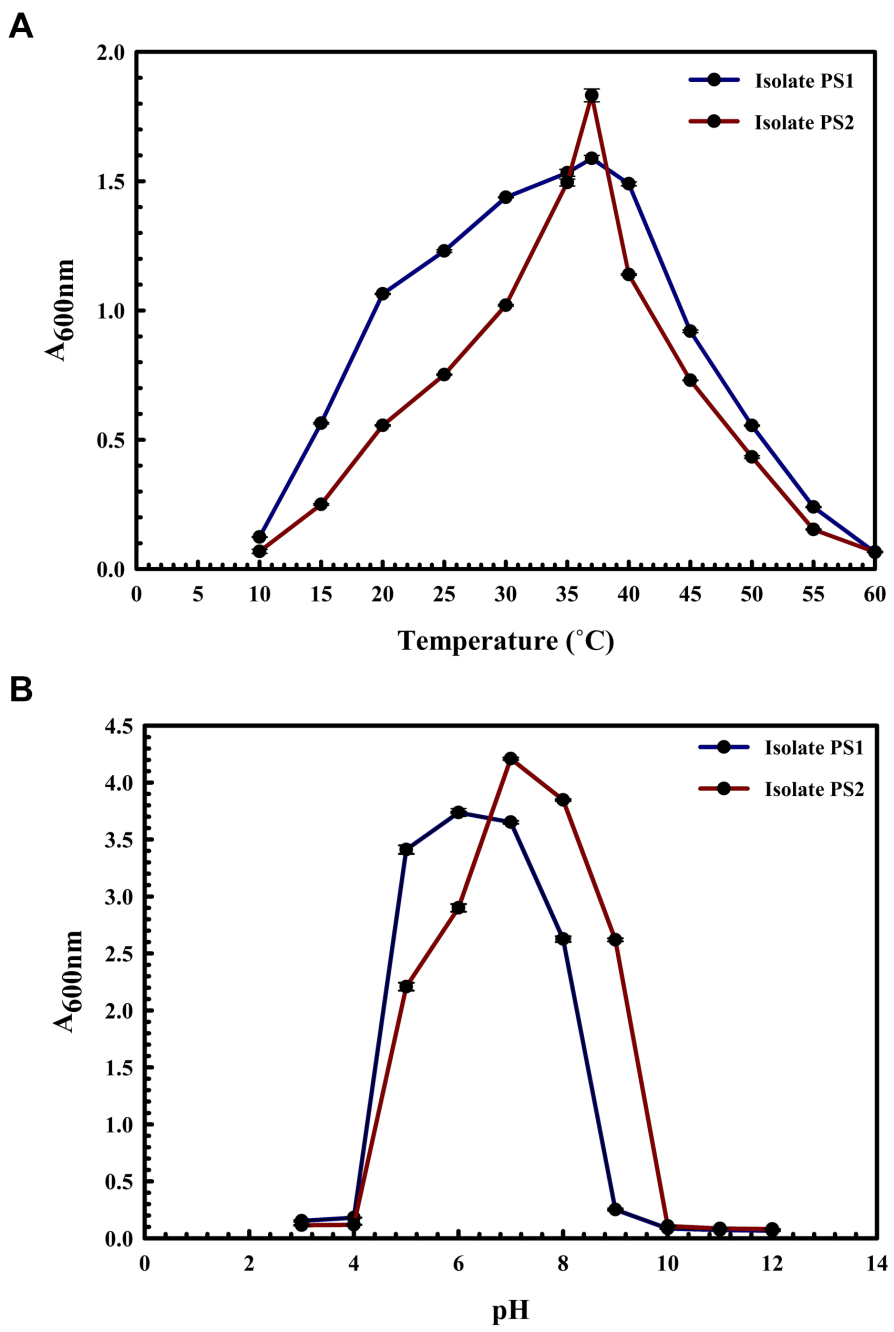
Growth pattern analysis of bacterial isolates at different temperatures and pH conditions. Growth was observed after incubating the cultures in LB broth with constant shaking at 200 rpm at temperatures ranging from 10°C to 60°C with an interval of 5°C **(A)** and pH from 3 to 12 with an interval of one **(B)**. The experiment was carried out in triplicates and growth was observed by taking absorbance at 600 nm. Plotted values are the mean of triplicate readings along with the observed standard deviation.

BLASTn analysis of the 16S rRNA gene sequences of PS1 (1,446 bp) and PS2 (1,429 bp) exhibited 98.62 and 98.46% similarity, with *P. agglomerans* NCTC 9381 and *P. agglomerans* ATCC 27155 in rRNA/ITS databases (NCBI). Phylogenetic analysis with 16S rRNA gene sequences of PS1 and PS2 with rRNA/ITS databases homologs further supported these findings (Figure 1). The 16S rRNA gene sequences of PS1 and PS2 showed a similarity of 99.48%, indicating their subspecies-level distinctiveness. Bacterial isolates PS1 and PS2 were designated as *P. agglomerans* PS1 and *P. agglomerans* PS2, respectively, for subsequent analyses. Microscopic investigation indicated *P. agglomerans* PS1 to be gram-negative, rod-shaped, and motile, while *P. agglomerans* PS2 was observed as gram-negative, rod-shaped, and non-motile. *P. agglomerans* PS1 showed optimum growth at 37°C (Figure 2A) within the pH range of 6.0–7.0 (Figure 2B). *P. agglomerans* PS2 showed optimum growth at pH 7.0 and 37°C (Figures 2A,B). Growth pattern analysis indicated that *P. agglomerans* PS1 and PS2 attain a log phase after 12.5 and 12 h, respectively. The doubling time of *P. agglomerans* PS1 (Supplementary Figure S1A) and PS2 (Supplementary Figure S1B) was calculated as 32.42 and 54.31 min, respectively. *P. agglomerans* PS1 showed 0.396 OD at 600 nm when grown anaerobically for 24 h at 37°C, indicating its facultative anaerobic nature. *P. agglomerans* PS2 did not show any growth under identical conditions and thus inferred as strict aerobe.

**Figure 3:**
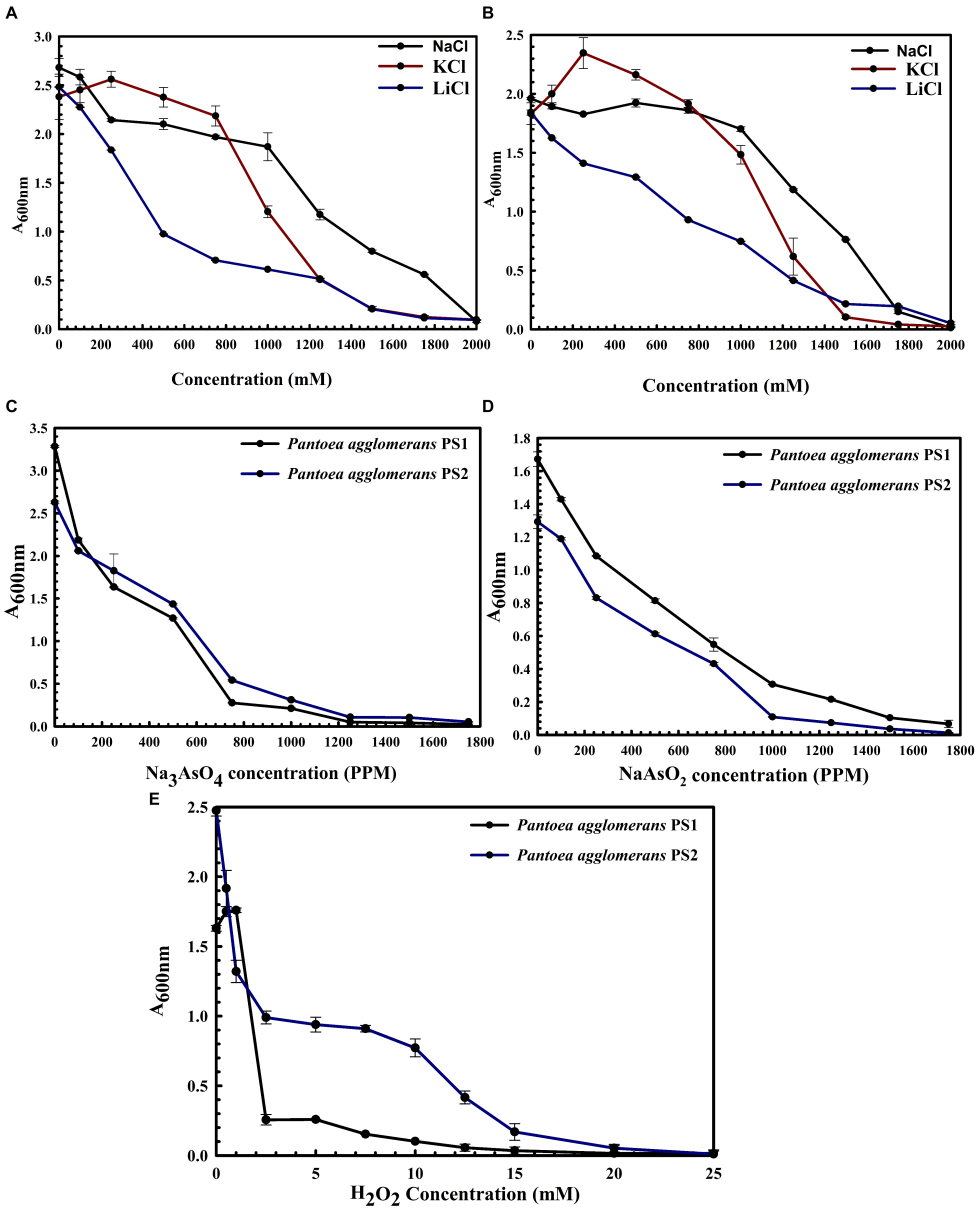
Growth pattern analysis of *Pantoea agglomerans* PS1 and PS2 in the presence of salts **(A,B)**, arsenic **(C,D)**, and hydrogen peroxide **(E)**. Growth pattern of PS1 **(A)** and PS2 **(B)** in saline conditions **(A)** was observed after incubating the cultures in LB broth supplemented with various salt concentrations (NaCl, KCl: LiCl) from 0, 250 mM, 500 mM, 750 mM, 1,000 mM, 1,250 mM, 1,500 mM, 1750 mM, 2000 mM at 37°C for 24 h with constant shaking at 200 rpm. Growth pattern PS1 and PS2 in the presence of sodium arsenate **(C)** and sodium arsenite **(D)** was observed after incubating the cultures in LB broth supplemented with different concentrations of sodium arsenite and sodium arsenate at 37°C for 24 h with constant shaking at 200 rpm. The growth pattern of PS1 and PS2 in the presence of hydrogen peroxide **(E)** was observed after incubating the cultures in LB broth supplemented with different hydrogen peroxide concentrations at 37°C for 24 h with constant shaking at 200 rpm. Experiments were carried out in triplicates and growth was observed by taking absorbance at 600 nm. Values plotted are the mean of triplicate readings along with the observed standard deviation.

*P. agglomerans* PS1 was positive for amylase, esterase, lipase, protease, and catalase activity, while *P. agglomerans* PS2 was positive for amylase, esterase, protease, and catalase activity. Substrate utilization assay of *P. agglomerans* PS1 and PS2 indicates their substrate utilization profile is similar to other *P. agglomerans* species (Supplementary Table S1). Antibiotic susceptibility assay indicated that *P. agglomerans* PS1 was resistant toward amikacin, novobiocin, cefotaxime, lincomycin antibiotics while showing sensitivity towards amoxicillin, cephalothin, erythromycin, oxytetracyclin, vancomycin, ceflnaxone, ceflazidime, netillin, ofloxacin and bacitracin (Supplementary Table S2). Similarly, *P. agglomerans* PS2 was found resistant towards amikacin, ceflnaxone, vancomycin, cephalothin while showing sensitivity towards novobiocin, cefotaxime, lincomycin, amoxicillin, erythromycin, oxytetracyclin, ceflazidime, netillin, ofloxacin, and bacitracin (Supplementary Table S2). The antibiotic resistance profile of *P. agglomerans* PS1 and PS2 was similar to other *Pantoea agglomerans* species (Supplementary Table S2). Similar biochemical, substrate utilization, and antibiotic resistance profiles of *P. agglomerans* PS1 and PS2 to other *Pantoea* species strengthen the 16S rRNA gene-based taxonomic observations. Stress response physiology indicated that *P. agglomerans* PS1 and PS2 can successfully grow in the presence of salts (Figures 3A,B), arsenic (Figures 3C,D), and oxidizing agents (Figure 3E), respectively, as observed for other *Pantoea* sp. (Supplementary Table S3).

### Genome characterisation of *Pantoea agglomerans* PS1 and PS2

Genome sequencing of *P. agglomerans* PS1 and PS2 resulted in 1,020,610 and 683,599 paired-end raw reads, respectively. *P. agglomerans* PS1 and PS2 reads were assembled into 96 and 406 contigs accounting for 4,987,053 bps and 5,177,646 bps, respectively (Supplementary Table S4). Functional annotation of the *P. agglomerans* PS1 genome identified 4,860 protein-coding sequences, 09 rRNA genes, 71 tRNA genes, and 01 tmRNA gene. *P. agglomerans* PS2 genome encoded 5,275 protein-coding sequences, 11 rRNA genes, and 74 tRNA genes. The average ANI among different species of *Pantoea* ranged from 74–99%, indicating significant interspecific genomic variations. Furthermore, the ANI scores of *P. agglomerans* PS1 and *P. agglomerans* PS2 with *P. agglomerans* strain AR1a and *P. agglomerans* CFSAN047153 were 99.59 and 99.83, respectively, which were comparatively higher than those with other members of the species (Supplementary Table S5). The affiliation of *P. agglomerans* PS1 and PS2 as a member of *P. agglomerans* species was further reconfirmed using terra correlation. *P.* sp. CFSAN033090 had been awarded 0.99951 z-score against both *P. agglomerans* PS1 and PS2 during terra-correlation, confirming their similarity with *P. agglomerans*. Other *Pantoea* species exhibited good similarity (z-score ∼0.95–0.99) (Supplementary Table S6).

**Figure 4:**
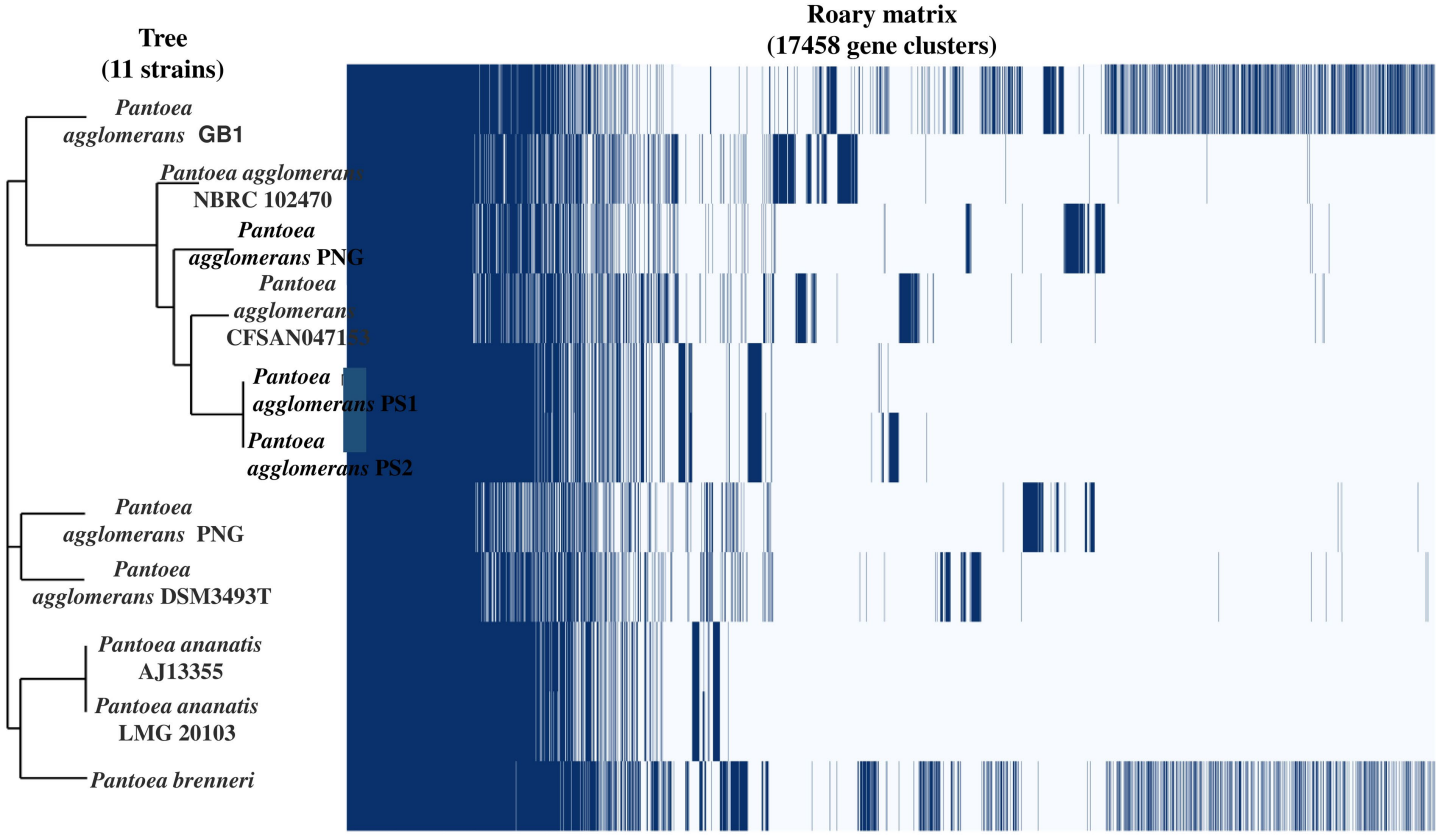
The phylogenomic tree constructed with the FastTree v2.1.10 tool via Roary. The left panel represents the phylogenetic relation of *Pantoea agglomerans* PS1 and PS2 with other *Pantoea* sp. The right panel depicts the core and accessory genes shared by different *Pantoea agglomerans* strains.

After the ANIb and tetra confirmation, the *P. agglomerans* PS1 and PS2 genomes were compared with genomes of *Pantoea* species to analyze genome-wide similarities and distinctiveness. The matrix generated using the Roary tool showed the comprehensive nature of the genome in which the *P. agglomerans* PS1 and PS2 showed the highest similarity with *Pantoea* sp. CFSAN033090 (Figure 4). It also revealed that all *Pantoea* genomes share only a few numbers of genes as their core genome. Shell and cloud genome collectively forms the central part of the genomes. *P. agglomerans* PS1 and PS2 genome has neither a pathogenic gene/island nor any virulence-related genes, indicating their non-pathogenic behavior. The genomic surveillance of both microbes revealed the presence of genes for phosphate solubilization and transport (Table 1). In addition to the genes for phosphate solubilization, *P. agglomerans* PS1 and PS2 genomes harbor genes encoding proteins for plant growth promotion activities like auxin biosynthesis, nitrogen assimilation, and siderophore biosynthesis (Table 2). *P. agglomerans* PS1 and PS2 genomes also harbor genes responsible for arsenic resistance, oxidative stress tolerance, metal stress tolerance, and salt tolerance (Supplementary Table S7) explaining its stress response physiology. The thirty-eight CAZymes clusters in the *P. agglomerans* PS1 genome (Supplementary Table S8) and twenty-nine CAZymes clusters within the *P. agglomerans* PS2 (Supplementary Table S9) genome indicate their diverse carbohydrate utilization profile. Several proteins were identified to be essential for effective colonization in plant rhizosphere (Kumar et al., 2023). An in-depth analysis of *the P. agglomerans* PS1 and PS2 genomes identifies the presence of genes encoding proteins for the synthesis of Type 1 & IV pili, exopolysaccharide (Table 3) essential for plant surface adhesion, auto-aggregation, and early biofilm formation (Kumar et al., 2023).

**Table 1:** Genetic features identified in *Pantoea agglomerans* PS1 *and* PS2 genomes associated with phosphate transport and solubilizing activity.

CDS start Position in the genome	CDS stop position in the genome	Strand	CDS start Position in the genome	CDS stop position in the genome	Strand	Function
*P. agglomerans* PS1			*P. agglomerans* PS2			
208,896	208,162	−	155,008	154,274	−	Phosphate transport system regulatory protein PhoU
209,687	208,914	−	155,799	155,026	−	Phosphate ABC transporter, ATP-binding protein PstB
210,622	209,732	−	156,734	155,844	−	Phosphate ABC transporter, permease protein PstA
211,581	210,619	−	157,693	156,731	−	Phosphate ABC transporter, permease protein PstC
212,712	211,669	−	158,824	157,781	−	Phosphate ABC transporter, substrate-binding protein PstS
485,460	484,540	−	−	−		Phosphate ABC transporter, substrate-binding protein PstS
486,793	485,480	−	103,243	103,932	+	Phosphate regulon sensor protein PhoR (SphS)
487,497	486,808	−	103,947	105,260	+	Phosphate regulon transcriptional regulatory protein PhoB (SphR)
192,619	194,373	+	489,852	491,606	+	Inner membrane protein YejM, alkaline phosphatase superfamily
54,568	55,581	+	54,362	55,375	+	Alkaline phosphatase isozyme conversion protein
79,170	78,286	−	241,638	240,754	−	Phosphonate ABC transporter permease protein PhnE1
80,027	79,167	−	242,495	241,635	−	Phosphonate ABC transporter permease protein PhnE2
81,047	80,121	−	243,515	242,589	−	Phosphonate ABC transporter substrate-binding protein PhnD
81,904	81,074	−	244,372	243,542	−	Phosphonate ABC transporter ATP-binding protein PhnC
82,823	81,984	−	245,291	244,452	−	Metal-dependent hydrolases of the beta-lactamase superfamily I; PhnP protein
83,356	82,820	−	245,824	245,288	−	Ribose 1,5-bisphosphate phosphokinase PhnN
84,492	83,356	−	246,960	245,824	−	Alpha-D-ribose 1-methylphosphonate 5-triphosphate diphosphatase
85,202	84,489	−	247,670	246,957	−	Alpha-D-ribose 1-methylphosphonate 5-triphosphate synthase subunit PhnL
85,964	85,203	−	248,432	247,671	−	Phosphonates utilization ATP-binding protein PhnK
86,802	85,954	−	249,270	248,422	−	Alpha-D-ribose 1-methylphosphonate 5-phosphate C-P lyase
87,871	86,795	−	250,339	249,263	−	Alpha-D-ribose 1-methylphosphonate 5-triphosphate synthase subunit PhnI
88,446	87,871	−	250,914	250,339	−	Alpha-D-ribose 1-methylphosphonate 5-triphosphate synthase subunit PhnH
88,892	88,446	−	251,360	250,914	−	Alpha-D-ribose 1-methylphosphonate 5-triphosphate synthase subunit PhnG
89,605	88,892	−	252,073	251,360	−	Transcriptional regulator PhnF
485,460	484,540	−	−	−	−	Phosphate ABC transporter, substrate-binding protein PstS (TC 3.A.1.7.1)
486,793	485,480	−	−	−	−	Phosphate regulon sensor protein PhoR (SphS) (EC 2.7.13.3)
487,497	486,808	−	−	−	−	Phosphate regulon transcriptional regulatory protein PhoB (SphR)
174,236	172,776	−	765,049	763,589	−	Sensor histidine kinase PhoQ (EC 2.7.13.3)
174,907	174,239	−	765,720	765,052	−	Transcriptional regulatory protein PhoP
256,331	256,924	+	257,030	257,623	+	Hexose-phosphate uptake two-component transcriptional response regulator UhpA
256,921	258,420	+	257,620	259,119	+	Hexose-phosphate uptake signal transduction histidine-protein kinase/phosphatase UhpB
258,430	259,758	+	259,129	260,457	+	Periplasmic space glucose-6-phosphate sensor protein UhpC
259,911	261,302	+	260,610	262,001	+	Hexose phosphate transport protein UhpT

**Table 2:** Genetic features identified within *Pantoea agglomerans* PS1 *and* PS2 genome encoding various proteins involved in nutrient assimilation.

CDS start Position in the genome	CDS stop position in the genome	Strand	CDS start Position in the genome	CDS stop position in the genome	Strand	Function
*P. agglomerans* PS1			*P. agglomerans* PS2			
A. IAA Biosynthesis						
138,108	139,067	+	435,341	436,300	+	Auxin efflux carrier family protein
5,911	7,272	+	42,302	43,663	+	Indole-3-glycerol phosphate synthase (EC 4.1.1.48) / Phosphoribosylanthranilate isomerase (EC 5.3.1.24)
76,788	76,318	−	96,718	97,188	+	Indole pyruvate decarboxylase (EC 4.2.1.122)
B. Nitrogen transport and regulation						
424,732	426,420	+	197,994	199,682	+	Nitrate/nitrite sensor protein NarQ
426,558	427,187	+	199,820	200,449	+	Nitrate/nitrite response regulator protein NarP
31,351	30,674	−	31,145	30,468	−	Respiratory nitrate reductase gamma chain (EC 1.7.99.4)
32,076	31,348	−	31,870	31,142	−	Respiratory nitrate reductase delta chain (EC 1.7.99.4)
33,617	32,073	−	33,411	31,867	−	Respiratory nitrate reductase beta chain (EC 1.7.99.4)
37,375	33,614	−	37,169	33,408	−	Respiratory nitrate reductase alpha chain (EC 1.7.99.4)
38,868	37,477	−	38,662	37,271	−	Nitrate/nitrite transporter NarK/U
39,185	39,832	+	38,979	39,626	+	Nitrate/nitrite response regulator protein NarL
324,380	325,633	+	915,193	916,446	+	Nitrate ABC transporter, substrate-binding protein
325,645	326,517	+	916,458	917,330	+	Nitrate ABC transporter, permease protein
326,528	327,316	+	917,341	918,129	+	Nitrate ABC transporter, ATP-binding protein
327,327	331,391	+	59,183	61,723	+	Nitrite reductase [NAD(P)H] large subunit (EC 1.7.1.4)
331,388	334,006	+	1	693	+	Assimilatory nitrate reductase large subunit (EC 1.7.99.4)
322,906	324,120	+	913,719	914,933	+	Response regulator NasT
324,380	325,633	+	915,193	916,446	+	Nitrate ABC transporter, substrate-binding protein
325,645	326,517	+	916,458	917,330	+	Nitrate ABC transporter, permease protein
326,528	327,316	+	917,341	918,129	+	Nitrate ABC transporter, ATP-binding protein
327,327	331,391	+	918,140	922,204	+	Nitrite reductase [NAD(P)H] large subunit (EC 1.7.1.4)
331,388	334,006	+	922,201	924,819	+	Assimilatory nitrate reductase large subunit (EC 1.7.99.4)
426,420	+	−	197,994	199,682	+	Nitrate/nitrite sensor protein NarQ
427,187	+	−	199,820	200,449	+	Nitrate/nitrite response regulator protein NarP
524,828	524,490	−	298,090	297,752	−	Nitrogen regulatory protein P-II
428,705	428,367	−	162,035	162,373	+	Nitrogen regulatory protein P-II, GlnK
11,639	10,230	−	11,639	10,230	−	Nitrogen regulation protein NR(I), GlnG (=NtrC)
12,696	11,647	−	12,696	11,647	−	Nitrogen regulation protein NtrB (EC 2.7.13.3)
2,261	1,344	−	299,494	298,577	−	Nitrogen assimilation regulatory protein Nac
−	−	−	61,720	62,046	+	Nitrite reductase [NAD(P)H] small subunit (EC 1.7.1.4)
179,553	179,074	−	429,172	429,651	+	PTS IIA-like nitrogen-regulatory protein PtsN
C. Siderophore biosynthesis						
9,318	11,480	+	201,288	203,510	+	Putative OMR family iron-siderophore receptor precursor
195,239	195,967	+	492,472	493,200	+	siderophore biosynthesis protein, putative
197,776	196,124	−	495,009	493,357	−	ABC-type siderophore export system, fused ATPase and permease components
127,695	125,503	−	198,853	196,661	−	Outer Membrane Siderophore Receptor IroN
130,130	132,352	+	201,288	203,510	+	Putative OMR family iron-siderophore receptor precursor
149,678	147,324	−	220,836	218,482	−	Siderophore [Alcaligin-like] biosynthesis complex, long chain @ Siderophore synthetase component, ligase
150,972	149,680	−	222,130	220,838	−	Siderophore [Alcaligin-like] biosynthetic enzyme (EC 1.14.13.59) @ Siderophore biosynthesis protein, monooxygenase
152,536	150,983	−	223,694	222,141	−	Siderophore [Alcaligin-like] decarboxylase (EC 4.1.1.-) @ Siderophore biosynthesis L-2,4-diaminobutyrate decarboxylase
196,845	195,991	−	411,880	412,734	+	Isochorismatase (EC 3.3.2.1) of siderophore biosynthesis
−	−	−	3	653	+	TonB-dependent siderophore receptor
−	−	−	2	517	+	Siderophore biosynthesis non-ribosomal peptide synthetase modules
−	−	−	386	3	−	Outer membrane (iron.B12.siderophore.hemin) receptor

**Table 3:** Genetic features within the genome of *Pantoea agglomerans* PS1 and PS2 encoding proteins for colonization in wheat rhizosphere.

CDS start Position in the genome	CDS stop position in the genome	Strand	CDS start Position in the genome	CDS stop position in the genome	Strand	Function
*P. agglomerans* PS1			*P. agglomerans* PS2			
A. Pili formation Protein						
139,693	138,677	−	468,517	469,035	+	P pilus assembly protein, pilin FimA
140,208	139,690	−	469,032	470,048	+	P pilus assembly protein, pilin FimA
142,727	140,199	−	465,998	468,526	+	Outer membrane usher protein fimD precursor
103,625	104,080	+	104,324	104,779	+	Type IV pilin PilA
104,067	105,452	+	104,766	106,151	+	Type IV fimbrial assembly, ATPase PilB
105,445	106,644	+	106,144	107,343	+	Type IV fimbrial assembly protein PilC
290,293	293,094	+	290,087	292,888	+	Type IV secretory pathway, VirB4 components
499,711	498,926	−	272,973	272,188	−	Type IV pilus biogenesis protein PilF
71,150	69,990	−	71,150	69,990	−	Type IV pilus biogenesis protein PilQ
73,408	72,602	−	73,408	72,602	−	Type IV pilus biogenesis protein PilM
−	−	−	488	126	−	Type IV pilus biogenesis protein PilZ
B. Mannose-6-phosphate isomerase						
435,620	435,309	−	506,778	506,467	−	Mannose-6-phosphate isomerase
274,861	276,036	+	1,096,846	1,095,671	−	Mannose-6-phosphate isomerase (EC 5.3.1.8)
C. EPS biosynthesis						
19,114	20,580	+	19,114	20,580	+	Exopolysaccharide biosynthesis polyprenyl glycosylphosphotransferase (IPR017475)
129,064	128,822	−	−	−	−	Exopolysaccharide synthesis ExoD

### Assessment of phosphate solubilizing activity of *Pantoea agglomerans* PS1 and PS2

*P. agglomerans* PS1 and PS2 were observed to grow on Pikovskaya agar plates, which was further validated through quantitative phosphatase assay. *P. agglomerans* PS1 exhibited extracellular alkaline phosphatase (420.51 IU) and acid phosphatase activity (57 IU), while *P. agglomerans* PS2 displayed extracellular alkaline phosphatase (476.089 IU) and acid phosphatase activity (63 IU). Similarly, *P. agglomerans* PS1 and PS2 showed intracellular alkaline phosphatase (22 IU and 15 IU) and acid phosphatase activity (10.1 IU and 6.5 IU), respectively.

### The plant growth promotion potential of *Pantoea agglomerans* PS1 and PS2

*P. agglomerans* PS1 and PS2 exhibited nitrate reductase activity (486 IU and 526 IU) and produced IAA (0.671 IU and 0.725 IU), respectively. The qualitative assessment of ammonia-producing activity indicated that both strains were involved in ammonia production. Siderophore biosynthesis assay indicated the appearance of an orange color and zones 22 ± 0.058 mm and 17 ± 0.025 mm for *P. agglomerans* PS1 and PS2, respectively. These results indicated the siderophore biosynthesis property of *P. agglomerans* PS1 and PS2. Furthermore, they were capable of producing and secreting plant growth-promoting hormones into the surrounding environment. The presence of genes responsible for plant growth promotion and their demonstrated bioactivity suggested that *P. agglomerans* PS1 and PS2 possess nutrient assimilation properties to boost plant growth.

### Assessment of drought and oxidative stress tolerance ability of microbial isolates

**Figure 5:**
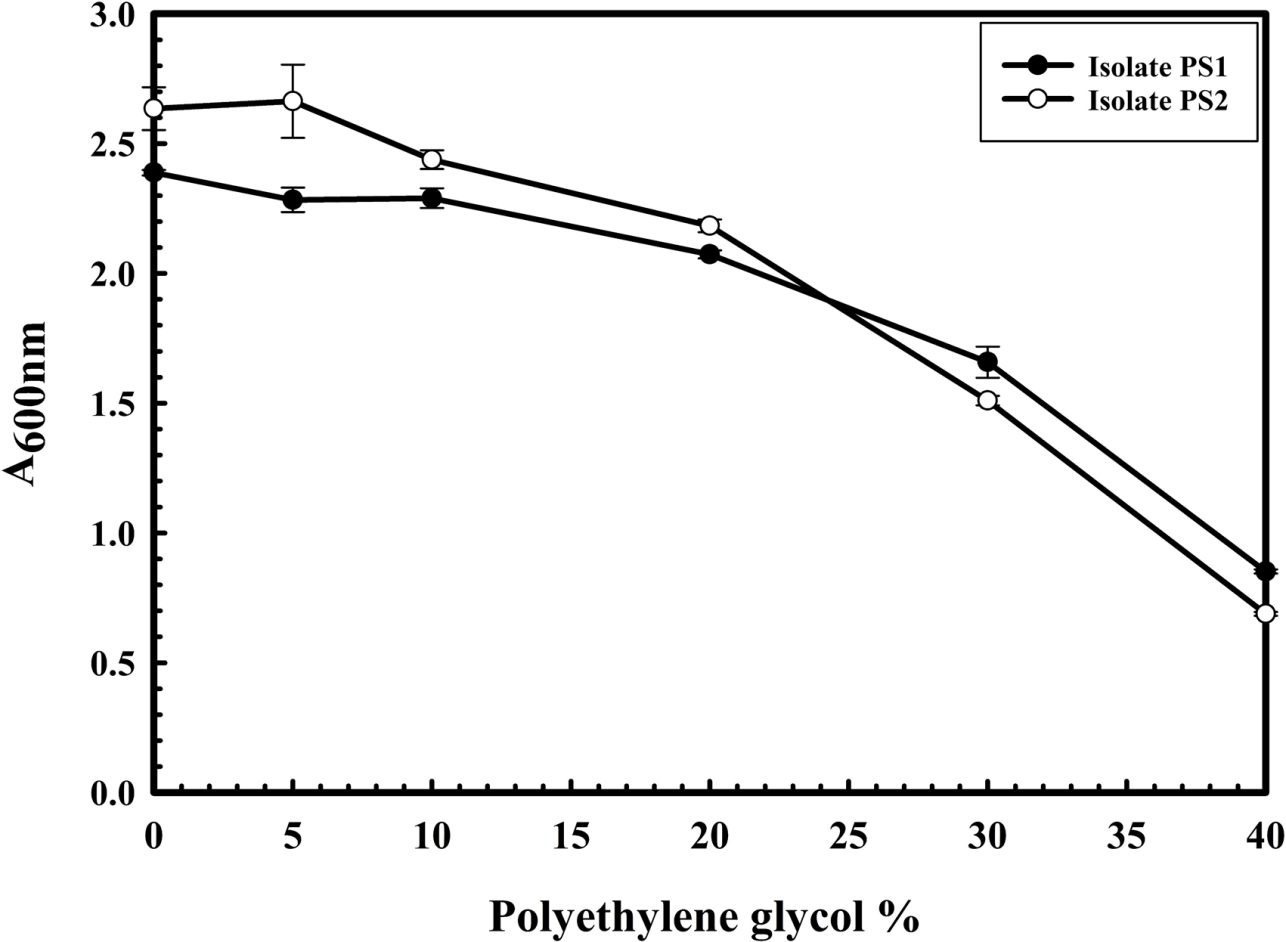
The drought stress tolerance ability of *Pantoea agglomerans* PS1 and PS2. Bacterial growth was observed after incubating the cultures in nutrient broth supplemented with different PEG concentrations at 37°C for 24 h with constant shaking at 200 rpm. The experiment was carried out in triplicates and growth was observed by taking absorbance at 600 nm. Values plotted are the mean of triplicate readings along with the observed standard deviation.

The microbial isolates PS1 and PS2 exhibited robust growth up to 40% of polyethylene glycol (PEG) concentration. Interestingly, PS2 showed greater activity compared to PS1. These findings highlight the plant growth-promoting features under drought stress (Figure 5). Microbial isolate PS2 (0.532 EU) was found to produce more ACC deaminase enzyme as compared to PS1 (0.436 EU). ACC deaminase activity in both strains confirms their oxidative stress-mitigating properties.

### Influence of *Pantoea agglomerans* PS1 and PS2 on seed germination under salt stress conditions

**Figure 6:**
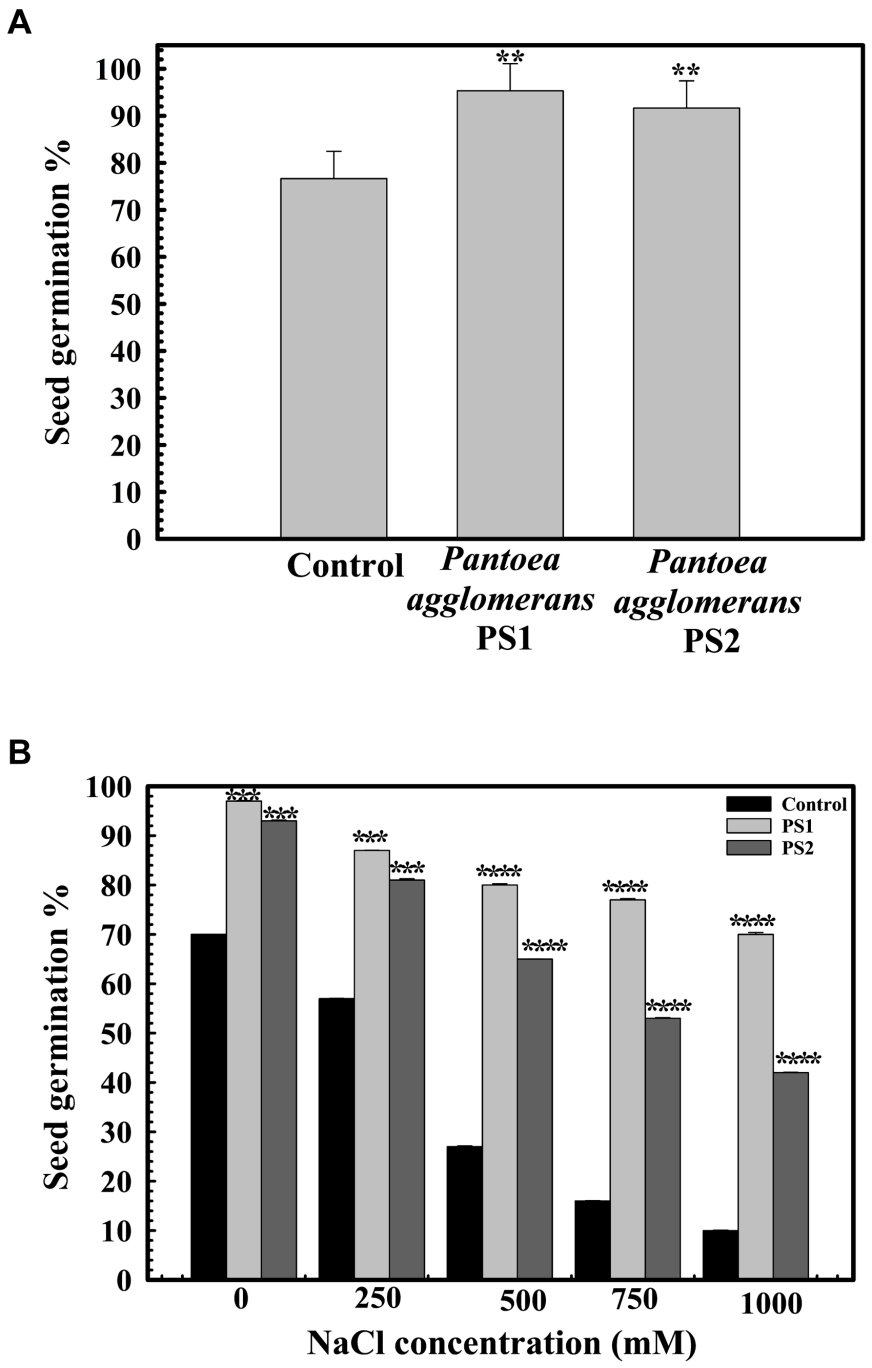
Influence of *Pantoea agglomerans* PS1 and PS2 on seed germination during normal **(A)** and saline conditions **(B)**. Seeds were inoculated in 2× 10^8^ CFU/mL of bacterial isolate. All assays were performed in triplicates. Statistical significance was calculated by comparing the observations of the bacterial pre-inoculated dataset against the observations of the control dataset. Here **** means the calculated *p*-value is >0.0001, *** means the calculated *p*-value is >0.001, and ** means the calculated *p-*value is >0.01.

A seed germination rate of 70.66% ± 0.57735 was observed in the control group. However, seeds pre-treated with *P. agglomerans* PS1 and PS2 exhibited germination efficiencies of 95.6 ± 0.57735 and 94% ±0.57705%, respectively (Figure 6A). *P. agglomerans* PS1 and PS2 were found to increase seed germination by ~135-and ~ 133-fold, respectively. Furthermore, pre-treatment with *P. agglomerans* PS1 and PS2 not only enhanced seed germination but also significantly improved it compared to the control (*p* < 0.001). Pre-treatment with *P. agglomerans* PS1 and PS2 also significantly increased alpha-amylase activity (0.956 IU (*p* = 0.0001) and 0.94 IU (*p* = 0.0001)), respectively, compared to the control (0.253 IU). The significant increase in alpha-amylase activity in wheat seeds after pre-treatment with *P. agglomerans* PS1 and PS2 could be a possible factor for enhanced seed germination. Seed germination significantly decreased (*p* = 0.020) with increasing salt concentration. *P. agglomerans* PS1 and PS2 pretreatment also exhibited enhanced seed germination under high salinity conditions (*p* = 0.004) (Figure 6B). PS2 not only improved seed germination but also promoted the growth of wheat plantlets. Wheat seeds pre-treated with *P. agglomerans* PS1 and PS2 demonstrated significantly increased shoot length (*p* = 0.001) and root length (*p* = 0.0018) compared to untreated seeds under high salinity conditions.

### Wheat rhizosphere microbiota profiling

**Figure 7:**
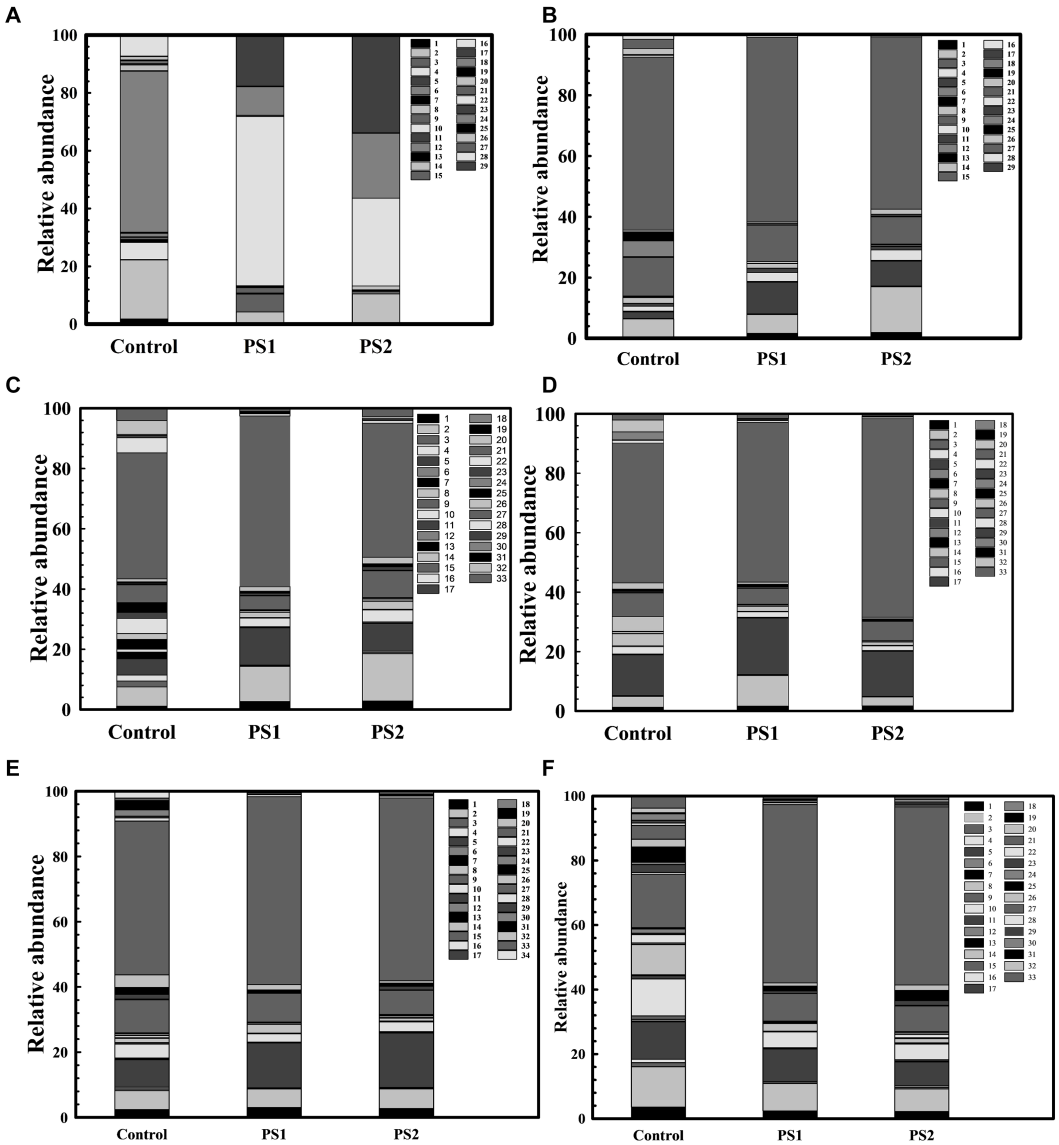
Wheat Rhizosphere microbiota composition in *Pantoea agglomerans* PS1 and PS2 inoculated and non-inoculated plants at different feeks [1.0 **(A)**, 2.0 **(B)**, 3.0 **(C)**, 6.0 **(D)**, 9.0 **(E)**, 10.5 **(F)**]. Here 1–34 represents different phyla represented as 1: Acidobacteria, 2: Actinobacteria, 3: Aquificae, 4: Armatimonadetes, 5: Bacteroidetes, 6: Caldiserica, 7: Chlamydiae, 8: Chlorobi, 9: Chloroflexi, 10: Chordata, 11: Chrysiogenetes, 12: Cyanobacteria, 13: Deferribacteres, 14: Deinococcus-Thermus, 15: Elusimicrobia, 16: Euryarchaeota, 17: Fibrobacteres, 18: Firmicutes, 19: Fusobacteria, 20: Gemmatimonadetes, 21: Nitrospirae, 22: Planctomycetes, 23: Spirochaetes, 24: Proteobacteria, 25: Synergistetes, 26: Tenericutes, 27: Thermodesulfobacteria, 28: Thermotogae, 29: Verrucomicrobia, 30: Candidatus Cloacimonetes, 31: Cyanobacteria, 32: Dictyoglomi, 33: Ignavibacteriae, 34: Thaumarchaeota.

**Figure 8:**
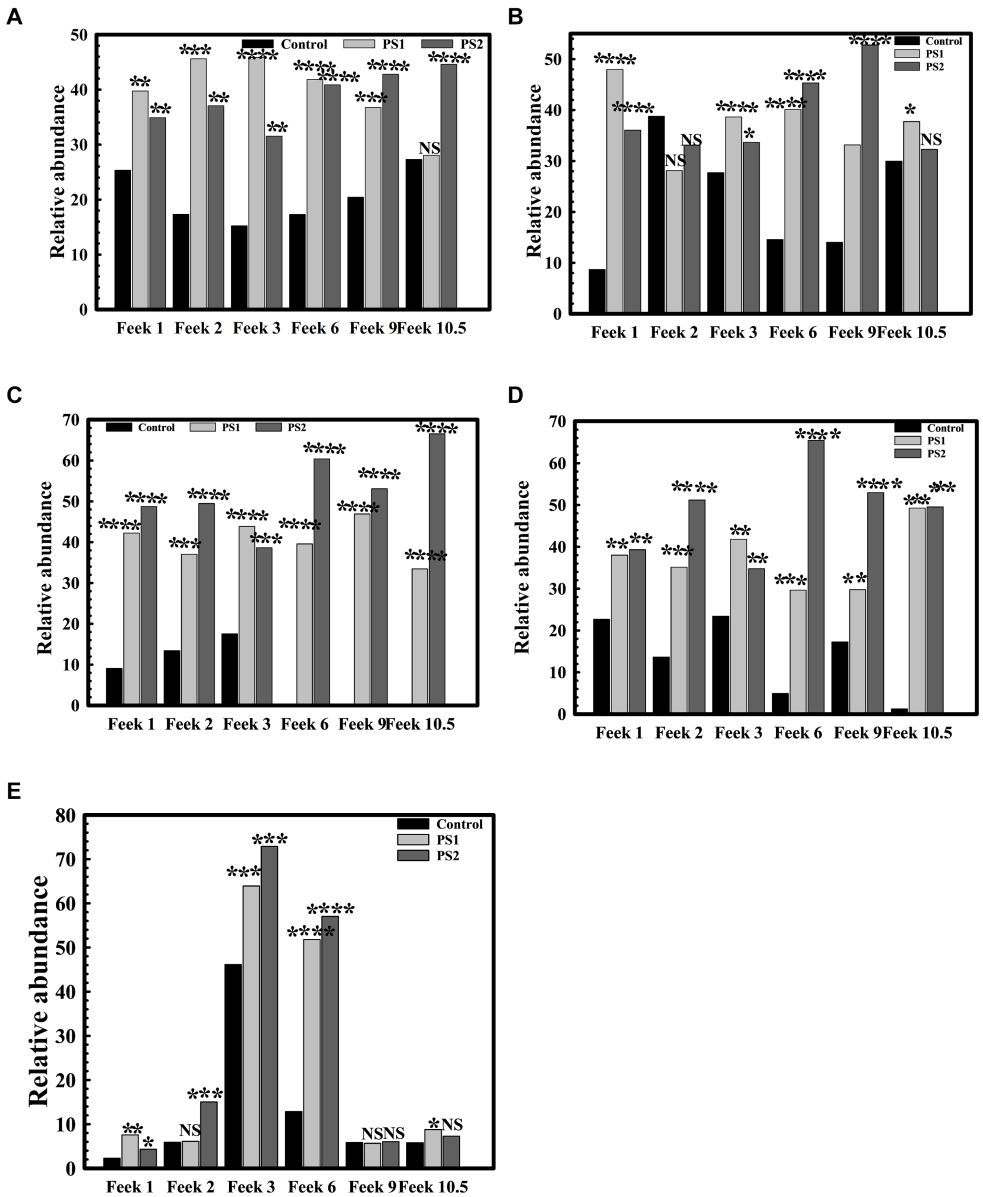
Relative abundance of Proteobacteria and Actinobacteria at different feeks in microbial inoculated and non-inoculated conditions. Relative abundance of Alphaproteobacteria **(A)**, Betaproteobacteria **(B)**, Gammaproteobacteria **(C)** Deltaproteobacteria **(E)** within Proteobacteria in bacterial inoculated and non-inoculated plants at different feeks (1.0, 2.0, 3.0, 6.0, 9.0, 10.5). Relative abundance of Actinobacteria among total microbial diversity in bacterial inoculated and non-inoculated plants at different feeks (1.0, 2.0, 3.0, 6.0, 9.0, 10.5) **(D)**. Statistical significance was calculated by comparing the observations of the bacterial pre-inoculated dataset against the observations the control dataset. Here **** means the calculated *p*-value is >0.0001, *** means the calculated *p*-value is >0.001, ** means the calculated *p-*value is >0.01, and * means the calculated *p-*value is >0.05.

The 16S rRNA gene analysis of wheat rhizosphere at different growth stages revealed variations in the microbiota with wheat growth stages (Figures 7A–F; Supplementary Table S10). At Feeks 1.0 (emergence stage), Alphaproteobacteria (Figure 8A), Betaproteobacteria (Figure 8B), and Gammaproteobacteria (Figure 8C) were abundant microbial groups in treated and non-treated wheat seedlings. However, the abundance of these microbial groups in treated seedlings was significantly (*p* < 0.001), different from non-treated ones (Figure 7). Among all bacterial groups, *Pantoea* sp. were significantly abundant (*p* < 0.001) in treated seeds with PS1 and PS2 than the non-treated ones. At Feeks 2.0 (beginning of tillering), rhizosphere microbiota primarily comprised Alphaproteobacteria (12.48% in PS1, 12.12% in PS2 and 6.47% in not-treated plants) (Figure 8A) Betaproteobacteria (Figure 8B) (34.35% in PS1, 32.42% in PS2 and 26% in not-treated plants) and Gammaproteobacteria (Figure 8C) (20.04% in PS1, 22% in PS2 and 9.45% in not-treated plants). The 16S rRNA gene sequences taxonomically affiliated to *Pantoea* sp. were observed in both treated as well as non-treated groups. However, 16S rRNA gene sequences taxonomically affiliated to *Pantoea* sp. were significantly higher (*p < 0.001*) in treated (0.38% in PS1 and 0.55% in PS2) than in the non-treated ones (0.15%).

At Feeks 3.0, Actinobacteria (46.15% in non-treated and 63.92 and 72.92% in PS1 and PS2 treated, respectively) (Figure 8D) was observed as a highly abundant microbial group, followed by Alphaproteobacteria (Figure 8A) (12.15% in non-treated, 15.36% in PS1 treated, and 18.31% PS2 treated), Betaproteobacteria (Figure 8B) (31.09% in non-treated, 38.52% in PS1 treated and 31.52% in PS2 treated plants), Deltaproteobacteria (Figure 8E) (15.16% in non-treated, 19% in PS1 treated, and 21.64% in PS2 treated), and Gammaproteobacteria (Figure 8C) (11.96% in non-treated, 26.4% in PS1 treated, and 26.55% in PS2 treated). Despite similar microbial abundance profiles, PS1 and PS2 showed significantly different (*p* < 0.01) rhizosphere microbiota compared to the untreated ones. Likewise, microbiota profiling at Feeks 2, the 16S rRNA gene sequences affiliated to *Pantoea* species were significantly abundant (*p* < 0.001) in PS1 (2.14%) and PS2 (2.47%) in comparison to untreated plants (0.16%). Additionally, *Flavobacteria* (1.95 and 4.15%), *Clostridia* (2.98, 4.33%)*, Chitinophagia* (4.7 and 4.31%), and *Bacilli* (3 and 4.7%) were exclusively associated with plants treated with *Pantoea agglomerans* PS1 and PS2, respectively.

At Feeks 6.0 (internode formation), Actinobacteria (12.84% in non-treated and 51.8, 57.06% in PS1 and PS2 treated) (Figure 8D) was the most abundant bacterial group, followed by, Alphaproteobacteria (16.03% in non-treated, 17.16% in PS1 treated, and 22.9% in PS2 treated) (Figure 8A), Betaproteobacteria (11.24% in non-treated, 22.72% in PS1 treated and 22.42% in PS2 treated) (Figure 8B), Deltaproteobacteria (0.003, 4.18 and 6.52% in non-treated, PS1, and PS2 treated respectively) (Figure 8E), and Gammaproteobacteria (1.43% in non-treated, 15.08% in PS1 treated and 19.74% in PS2 treated) (Figure 8C). Among Gammaproteobacteria, the 16S rRNA gene sequences affiliated with *Pantoea* were present in plants inoculated with only PS1 (0.74%) and PS2 (1.92%). Moreover, *Chitinophagia*, *Deltaproteobacteria*, and *Flavobacteria* were specifically associated with plants treated with *Pantoea agglomerans* PS1 and PS2.

At Feeks 9.0 (ligule of flag leaf visible), rhizosphere microbiota is represented by Alphaproteobacteria (7.34% in non-treated, 14.22% in PS1 treated and 15.07% in PS2 treated) (Figure 8A), Betaproteobacteria (7.35% in control, 15.66% in PS1 treated and 19.83% in PS2 treated) (Figure 8B), Deltaproteobacteria (4.44% in PS1 treated and 5.73% in PS2 treated) (Figure 8E), and Gammaproteobacteria (2.588% in control, 8.60% in PS1 treated and 11.91% in PS2 treated) (Figure 8C). Despite similar microbial abundance profiles, PS1 and PS2 showed significantly different (*p* < 0.01) rhizosphere microbiota compared to the untreated ones. Additionally, 16S rRNA gene sequences affiliated with *Pantoea* sp. were lower in PS1 (0.62%) and PS2 (1.51%) treated plants.

Finally, at Feeks 10.5 (heading and flowering), rhizosphere microbiota is represented by Alphaproteobacteria (6.37% in control, 13.11% in PS1 treated, 19.20% in PS2 treated) (Figure 8A), Betaproteobacteria (4.56% in control, 12.62% in PS1 treated and 9.94% in PS2 treated) (Figure 8B), Deltaproteobacteria (3.44% in PS1 treated and 6.13% in PS2 treated) (Figure 8E), and Gammaproteobacteria (0.36% in control, 11.77% in PS1 treated and 9.90% in PS2 treated) were present (Figure 8C). The 16S rRNA gene sequences affiliated with *Pantoea* were still present in the case of plants treated with PS1 (0.54%) and PS2 (1.4%) only.

Soil microbiota composition was also assessed to unveil the influence of soil microbiota in the development of wheat rhizosphere microbiota during various wheat growth stages. Proteobacteria was the dominant microbial group observed across all the soil samples collected parallelly with wheat rhizosphere samples during different growth stages (Feeks 1.0, 2.0, 3.0, 6.0, 9.0, and 10.5). However, variability was observed in proteobacterial subgroups. Gammaproteobacteria were the most abundant (33.16%) in the soil sample collected during Feeks 1.0, while the soil sample collected at Feeks 2.0 was marked by a high presence of Betaproteobacteria (17.48%). During Feeks 3.0, Feeks 6.0, Feeks 9.0, and Feeks 10.5, Alphaproteobacteria were the dominant class in all soil samples with relative abundances of 18.55, 18.32, 22.97, and 21.5%, respectively.

### Plant growth promoting potential of *Pantoea agglomerans* PS1 and PS2 in experimental field conditions

**Figure 9:**
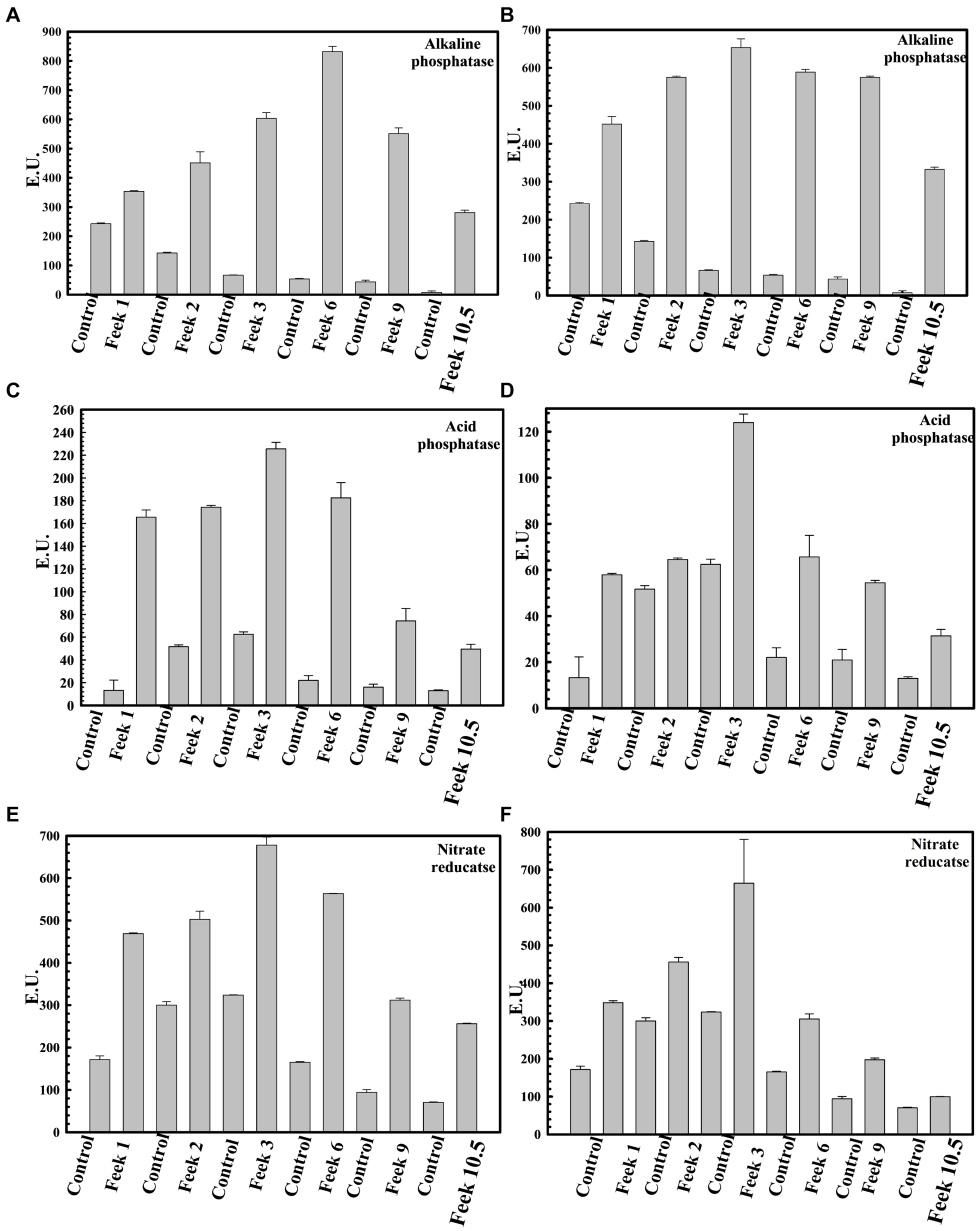
Assessment of plant growth promoting potential of *Pantoea agglomerans* PS1 and PS2 in field conditions. Alkaline phosphatase activity at different feeks (1.0, 2.0, 3.0, 6.0, 9.0, 10.5) in the PS1 **(A)** and PS2 **(B)** inoculated seeds in comparison to untreated seeds. Acid phosphatase activity at different feeks (1.0, 2.0, 3.0, 6.0, 9.0, 10.5) in the PS1 **(C)** and PS2 **(D)** inoculated seeds in comparison to untreated seeds. Nitrate reductase activity at different feeks (1.0, 2.0, 3.0, 6.0, 9.0, 10.5) in the PS1 **(E)** and PS2 **(F)** inoculated seeds in comparison to untreated seeds. Experiments were carried out in triplicate. Plotted values are the mean of triplicate readings along with their observed standard deviation.

Plant growth potential *P. agglomerans* PS1 and PS2 need to be also validated in field conditions to ensure their efficiency and suitability for agricultural application. Pre-treatment of seeds with *P. agglomerans* PS1 and PS2 showed a significant increase in the total sugar (13583.21 μg/g and 14490.5 μg/g) and reducing sugar (385 μg/g and 362.97 μg/g), respectively, at feeks 3.0 (Supplementary Figure S2). A respective increase in total sugar (10850.3 μg/g and 10301.5 μg/g) and reducing sugar content (321.7 μg/g and 300.73 μg/g) was observed in seeds treated with *P. agglomerans* PS1 and PS2 about control at feeks 6.0 (Supplementary Figure S2). Wheat plants treated with *P. agglomerans* PS1 showed an increase in extracellular alkaline phosphatase (831.83 IU) at feeks 3.0 (Figure 9A) and acid phosphatase (225.54 IU) (Figure 9C) activity at feeks 6.0, respectively. Similarly, *P. agglomerans* PS2 pre-treatment results in increased extracellular alkaline (653.29 IU) (Figure 9B) and acid phosphatase activity (123.9 IU) at feeks 3.0 (Figure 9D). *P. agglomerans* PS1 and *P. agglomerans* PS2 pre-treatment have also increased the nitrate reductase activity (678 IU and 664.5 IU) at feeks 3.0, respectively, compared to control (Figures 9E,F). Wheat plants treated with *Pantoea agglomerans* PS1 and PS2 showed a significant increase in the number of tillers (*p* = 0.0018), number of leaves per plant (*p* = 0.0001), spike length (*p* = 0.0001), number of spikes per plant (*p* = 0.0001), number of spikelets per plant (*p* = 0.0001), grain weight per 1,000 grains (*p* = 0.0023) and grain yield (*p* = 0.0001) (Table 4).

**Table 4:** Assessment of plant growth promoting potential of *Pantoea agglomerans* PS1 and PS2 in experimental field conditions.

Productivity phenotype	WC-306 Plants	WC-306 plants pre-inoculated with *P. agglomerans* PS1	WC-306 plants pre-inoculated with *P. agglomerans* PS2
Leaves per plant	12.34 ± 0.57	42 ± 1 (*p < 0.001*)	35.67 ± 0.257 (*p < 0.001*)
Number of tillers	3.34 ± 0.57	4.67 ± 0.57 (*p < 0.05*)	4 ± 0.71 (*p < 0.05*)
Number of spike/plant	26.3 ± 1.309	44.67 ± 1.52 (*p < 0.001*)	40.34 ± 1.03 (*p < 0.001*)
Spike length (cm)	15.46 ± 0.41	21.1 ± 0.96 (*p < 0.01*)	23.2 ± 0.46 (*p < 0.01*)
Number of spikelets per plant	30.34 ± 0.57	63.4 ± 1.52 (*p < 0.001*)	55.67 ± 0.152 (*p < 0.001*)
Grain weight (g)	30.23 ± 0.37	48 ± 0.21 (*p < 0.001*)	49.5 ± 0.07 (*p < 0.001*)
Grain yield (Kg/acre)	3,267	47044.8 (*p < 0.001*)	49,005 (*p < 0.001*)

All the experiments were performed in 10 biological replicates. Values shown in the table represent the mean value and observed standard deviation. Statistical significance was calculated by comparing the observations of the bacterial pre-inoculated dataset against the observations of WC306 plants.

## Discussion

Wheat is a prime source of energy for the majority of the population around the globe and its growth has to be uplifted to ensure food security for all (Shiferaw et al., 2013). The development of high-yielding pest-resistant varieties has paved the way to achieve it (Shiferaw et al., 2013). Now a strategy is required to fulfill elemental requirements for better plant growth and crop yield. Though our biosphere has an abundance of essential minerals for plant growth, however, their poor bioavailability limits their assimilation (Macik et al., 2020). Phosphorus is one of such essential elements and chemical fertilizers are being continuously used to fulfill plants’ growth requirements (Illakwahhi et al., 2024). Continuous usage of chemical fertilizers has severely affected soil ecology, thus soil fertility to support crop growth (Macik et al., 2020). Researchers are developing sustainable solutions to ensure plant growth requirements without impacting the soil ecology. Identification and application of biofertilizers seem to be one promising solution (Kumar et al., 2022). Plant rhizosphere microbiota is a potential source of the identification of promising biofertilizers (Aloo et al., 2022). In addition to extending plant growth-promoting properties, rhizospheric biofertilizers could also overcome application-related issues like stability, efficacy, and host specificity (Sharma et al., 2024).

Hereby, the present study was structured to explore wheat rhizosphere microbiota to identify potential phosphate-solubilizing biofertilizers to sustainably enhance crop yield to ensure food security. Microbial culturing and screening identified two promising phosphate-solubilizing bacterial isolates. Taxonomic, morphological, and physiological characterizations indicated their affiliation with *P. agglomerans.* Despite the similarity with the same species, varied physiological and genetic features indicated their diverse nature, hereby labeled as *P. agglomerans* PS1 and PS2. *P. agglomerans* have been identified from diverse plant-associated ecosystems including wheat rhizosphere (Links et al., 2014; Soluch et al., 2021). *P. agglomerans* strains were also characterized as plant endophytes and early colonizers (Remus et al., 2000). *P. agglomerans* strains were also known to enhance wheat biomass, indole acetic acid production, phosphate solubilization (Díaz Herrera et al., 2016), and priming host immune response (Ortmann and Moerschbacher, 2006). These studies suggest their potential as biofertilizers, however, *P. agglomerans* strains were also characterized as plant pathogens (Barash and Manulis-Sasson, 2009). It created a need for in-depth characterizations of identified strains before promoting them as biofertilizers. Biofertilizers have to be characterized for stability in dynamic soil ecosystems (Shah et al., 2022), plant growth-promoting properties (Sharma et al., 2024), stable colonization (Macik et al., 2020), and non-pathogenic behavior (Sharma et al., 2024). The stress response physiological analysis of both isolates indicates their survivability in varied pH (5–9), temperature (15–55°C), drought, and saline conditions [>10% (w/v)], as well as after exposure to arsenic. Their tolerance levels against these common soil stressors will ensure their performance as plant growth promoters. Studies reported the accumulation of various antibiotics in soil by the introduction of dung from farm animals or by human efforts such as discarded drugs, sludge, and effluent water (Cycoń et al., 2019) or soil microbes themselves (Hashmi et al., 2017). Both of our isolated microbes showed resistance against various antibiotics such as cefotaxime and lincomycin for *P. agglomerans* PS1 and amikacin, vancomycin, and ceflnaxone for *P. agglomerans* PS2 which was further confirmed by the presence of antibiotic resistance genes during genome-wide analysis. These findings suggested that these isolates could successfully overcome the antibiotic load added by human activities and antibiotics released by different microbes in their environment. The substrate utilization profile exhibits the adaptability of the microbes to utilize multiple carbon sources that provide them an advantage in environments with substrates other than their primary source (Wang et al., 2019; Sharma et al., 2024). These characterizations indicate their stability in a dynamic soil ecosystem, an essential biofertilizer property.

*P. agglomerans* PS1 and PS2 genomes indicated the presence of gene clusters for various biofertilizer properties like nutrient assimilation (Phosphate uptake and solubilization, nitrogen assimilation, siderophore biosynthesis), auxin biosynthesis, and colonization. Additionally, an in-depth exploration of *P. agglomerans* PS1 and PS2 genomes indicates the lack of plant pathogenesis-related genes. Functional assays also confirmed these biofertilizer properties of *P. agglomerans* PS1 and PS2. These observations are similar to the properties of *P. agglomerans* strains identified as potential biofertilizers (Links et al., 2014; Soluch et al., 2021). Salinity stress is one of the biggest bottlenecks in wheat crop yield. It induces ethylene production and suppresses plant growth (Afridi et al., 2019). ACC deaminase could overcome salinity-induced stress by restricting ethylene production and ensuring good crop yield (Afridi et al., 2019). Both *P. agglomerans* PS1 and PS2 showed an efficient ACC deaminase activity, indicating their plant growth promotion in salinity stress conditions. Translation of these plant growth properties to the host requires an in-depth assessment before confirming them as potential biofertilizers. Enhanced wheat seed germination with and without saline conditions after inoculation with *P. agglomerans* isolates, indicates plant growth promotion during early growth stages. These attributes could be due to the supplementation of amylase activity and auxin production by *P. agglomerans* PS1 and PS2. The α-amylase present in the aleurone layer hydrolyzes the endospermic starch and fulfills the energy requirement for the growth of root and shoot during seed germination (Kaneko et al., 2002). Experimental observations confirmed the amylase activity of *P. agglomerans* PS1 and PS2. Even amylase activity was found enhanced in wheat seeds inoculated *P. agglomerans* PS1 and PS2. These observations indicated that pre-inoculation seeds with *Pantoea agglomerans* PS1 and PS2 could have extended energy harvesting for effective seed germination. Auxins are well-known growth-regulating plant hormones that also determine morphogenesis in plants (George et al., 2007). These isolates could have extended an exogenous source of auxins and promoted the growth of the host plant. The plant growth promotion potential of *P. agglomerans* PS1 and PS2 was not only limited to seed germination but also significantly enhanced plant growth parameters during various growth stages. Preinoculation of wheat seeds with *P. agglomerans* PS1 and PS2 significantly enhanced plant biomass and crop yield. These results strongly indicated the biofertilizer potential of *P. agglomerans* PS1 and PS2 to sustainably enhance crop yield.

The stability of the biofertilizer strains during whole plant growth stages is another critical selection criterion (Macik et al., 2020). Additionally, the influence of microbial inoculation on plant rhizosphere microbiota also needs attention to define their mechanistic role in enhanced crop yield (Kong and Liu, 2022). Wheat rhizosphere microbiota exploration highlighted the stable presence of *P. agglomerans* PS1 and PS2 across all the growth stages. This implies the stability of *P. agglomerans* PS1 and PS2 over the developmental stages of wheat. Therefore, plant growth promotion properties of microbial strains are extended throughout plant growth stages. It could be a possible reason for better plant growth and yield in the treated group. *P. agglomerans-*specific 16S rRNA gene sequences were also observed in the first three growth stages in the untreated group, however, in very low abundance. *P. agglomerans* is a native member of the wheat rhizosphere (Links et al., 2014), so its presence in the untreated group is justified. Even *P. agglomerans* PS1 and PS2 were found to modulate the rhizosphere microbiota across wheat growth stages. The presence of *P. agglomerans* PS1 and PS2 was observed to recruit new members in the rhizosphere microbiota. Flavobacteriia, Clostridia, Chitinophagia, and Bacilli were exclusively abundant in treated plants at the tillering stage while Chitinophagia, Deltaproteobacteria, and Flavobacteriia were specifically associated with treated plants at the internode formation stage. Chitinophagia, Flavobacteriia, Clostridia, and Deltaproteobacteria have plant growth promotion characteristics. *Chitinophaga* exhibited plant growth-promoting (PGP) traits in different crops under stress and improves plant growth through the production of siderophores (Goswami and Deka, 2020). *Flavobacterium* were characterized to improve plant’s growth parameters such as water status, membrane integrity, osmolyte accumulation, stress response gene expression, and drought tolerance in wheat plants (Gontia-Mishra et al., 2016). *Clostridium* slowed down the colonization of roots by the fungal mycelium (Polianskaia et al., 2002). Recruitment of new members with plant growth promotion properties is known for better plant growth and production (Santoyo, 2022). Wheat plant growth parameters were found to change during the plant growth stages. These changes could be an outcome of restored rhizosphere microbiota. These findings develop an idea about the mechanistic role of *P. agglomerans* PS1 and PS2 in plant growth, however, in-depth explorations are required to establish it scientifically. These research findings confirm plant growth-promoting properties of *P. agglomerans* PS1 and PS2. Both strains could be utilized as efficient bio-fertilizers. This would be an economical and eco-friendly solution to mitigate the requirement of chemical fertilizers providing better availability of nutrients to plants.

## Data availability statement

The datasets presented in this study can be found in online repositories. The 16S rRNA gene sequence datasets generated in this study were deposited at NCBI with SRA accession ID PRJNA1136665 (https://www.ncbi.nlm.nih.gov/sra/PRJNA1136665). The whole genome sequence of P. agglomerans PS1 & PS2 has been uploaded to the NCBI server with SRA accession ID PRJNA1136672 (http://www.ncbi.nlm.nih.gov/bioproject/1136672).

## Author contributions

PS: Formal analysis, Investigation, Methodology, Visualization, Writing – original draft. RP: Data curation, Funding acquisition, Project administration, Resources, Supervision, Writing – review & editing. NC: Conceptualization, Project administration, Resources, Software, Supervision, Visualization, Writing – original draft.

## Footnotes

^1^ https://rast.nmpdr.org/rast.cgi?page=Jobs

^2^ https://github.com/sanger-pathogens/Roary/blob/master/contrib/roary_plots/roary_plots.py

^3^ https://www.sunflower.k-state.edu/agronomy/wheat/wheatdevelopment.html

^4^ https://www.genotypic.co.in/commander/
