# Supplementary material for: Unveiling wheat growth promotion potential of phosphate solubilizing Pantoea agglomerans PS1 and PS2 through genomic, physiological, and metagenomic characterizations
Source: Front Microbiol. 2024 Sep 9;15:1467082. doi: 10.3389/fmicb.2024.1467082 (PMC11420927; doi:10.3389/fmicb.2024.1467082)

Unveiling wheat growth promotion potential of phosphate solubilizing *Pantoea agglomerans* PS1 & PS2 through genomic, physiological, and metagenomic characterizations

Pinki Sharma[1], Rajesh Pandey[2,3], Nar Singh Chauhan[1*]

[1]Department of Biochemistry, Maharshi Dayanand University, Rohtak, Haryana, India
[2]INtegrative GENomics of HOst-PathogEn (INGEN-HOPE) laboratory, CSIR-Institute of Genomics and Integrative Biology (CSIR-IGIB), Mall Road, Delhi-110007, India.
[3]Academy of Scientific and Innovative Research (AcSIR), Ghaziabad-201002, India.

*Corresponding author
Rajesh Pandey (rajeshp@igib.res.in)
Nar Singh Chauhan (nschauhan@mdurohtak.ac.in)

Running Title: Wheat rhizospheric *Pantoea agglomerans* for sustainable agriculture

Number of Words:11387

Number of Figures: 9

Number of Tables: 4

Supplementary Table S1: Substrate utilization profile of *Pantoea agglomerans* PS1 and PS2 and their comparison with substrate utilization profile of phylogenetic-related *Pantoea* species.

No.	Test	*P. agglomerans* PS1	*P. agglomerans* PS2	*P. agglomerans* LMG 2660	*P. conspicua*	*P. dispersa* LMG 2603	*P. stewartii* LMG 2715	*P. stewartii* LMG 2632	*P. vagans* LMG 24199	*P. eucalypti* LMG 24197	*P.adeleyi* LMG 24200	*P. anthophila* LMG 2558	*P. ananatis* LMG 2665
1	Lactose	ND	ND	ND	+	ND	ND	+	ND	+	ND	ND	+
2	Xylose	+	+	+	+	+	+	+	+	+	+	+	+
3	Maltose	+	+	+	+	+	+	+	+	+	+	+	+
4	Fructose	ND	+	ND	ND	ND	ND	ND	ND	ND	ND	ND	ND
5	Dextrose	+	+	ND	ND	ND	ND	ND	ND	ND	ND	ND	ND
6	Raffinose	+	+	+	+	ND	+	+	ND	ND	ND	ND	+
7	Trehalose	+	ND	ND	ND	ND	ND	ND	ND	ND	ND	ND	ND
8<br>9	Melibiose	+	+	ND	ND	ND	+	+	ND	ND	ND	ND	+
10	Inulin	+	+	ND	ND	ND	ND	ND	ND	ND	ND	ND	ND
11	Glycerol	ND	+	ND	ND	ND	ND	ND	ND	ND	+	+	+
12	Dulcitol	+	ND	ND	+	ND	ND	ND	ND	ND	ND	ND	+
13	Inositol	ND	+	ND	+	ND	ND	+	+	+	ND	+	+
14	Sorbitol	ND	+	ND	ND	ND	ND	ND	ND	ND	ND	ND	+
15	Mannitol	ND	ND	ND	ND	ND	ND	+	ND	ND	ND	ND	ND
16	Adonitol	+	ND	ND	ND	ND	ND	ND	ND	ND	ND	ND	ND
17	Arabitol	ND	ND	ND	ND	ND	ND	+	ND	ND	ND	+	ND
18	Erythritol	ND	+	ND	ND	+	ND	ND	ND	ND	ND	ND	ND
19	Rhamnose	+	+	+	+	+	+	ND	+	+	+	+	+
20	Cellobiose	ND	+	ND	+			+				+	+
21	Xylitol	+	+	ND	ND	ND	ND	ND	ND	ND		ND	ND
22	ONPG	+	+	ND	+	ND	+	ND	ND	ND	+	ND	ND
23	Esculin hydrolysis	ND	ND	ND	ND	ND	ND	ND	+	ND	ND	ND	+
24	D-Arabinose	ND	ND	ND	ND	ND	+	ND	ND	ND	ND	ND	ND
25	Citrate utilization	+	+	ND	ND	ND	ND	ND	ND	ND	ND	+	+
26	Malonate utilisation	ND	ND	ND	ND	ND	ND	+	ND	ND	ND	ND	ND
27	Sorbose	+	+	ND	ND	+	ND	ND	ND	ND	ND	ND	ND

Here ND: Not defined in the literature.

Supplementary Table S2: Antibiotic susceptibility profile of *Pantoea agglomerans* PS1 and PS2 and their comparison with antibiotic susceptibility profile of phylogenetic-related *Pantoea* species.

**No.**	**Antibiotics**	***P. agglomerans* PS1**	***P. agglomerans* PS2**	***P. agglomerans* Sp.**	***P. conspicua***	***P. dispersa* LMG 2603**	***P.stewartii subsp. stewartii* LMG 2715**	***P. stewartii subsp. indologenes* LMG 2632**	***P. vagans* LMG 24199**	***P. eucalypti* LMG 24197**
1	Amikacin	+	+	+	+	ND	ND	ND	+	ND
2	Amoxicillin	ND	ND	ND	ND	+	ND	ND	ND	ND
3	Bacitracin	ND	ND	ND	ND	ND	+	+	ND	ND
4	Cephalothin	ND	+	ND	ND	ND	ND	ND	+	ND
5	Novobiocin	+	ND	ND	ND	ND	ND	+	ND	ND
6	Vancomycin	ND	+	ND	+	ND	+	ND	ND	ND
7	Ceflnaxone	ND	+	ND	+	ND	ND	ND	ND	ND
8	Ceflazidime	ND	ND	+	ND	ND	ND	ND	+	+
9	Cefotaxime	+	ND	+	ND	ND	ND	ND	+	+
10	Lincomycin	+	ND	ND	ND	ND	ND	ND	ND	ND
11	Ofloxacin	ND	ND	ND	ND	ND	ND	+	ND	ND
1	Amikacin	+	+	+	+	ND	ND	ND	+	ND
2	Amoxicillin	ND	ND	ND	ND	+	ND	ND	ND	ND
3	Bacitracin	ND	ND	ND	ND	ND	+	+	ND	ND

Supplementary Table S3: Growth parameters of *Pantoea agglomerans* PS1 and PS2 and their comparison with other *Pantoea* strains.

**Microbes**	**pH**	**Temperature (˚C)**	**Minimum Inhibitory Concentration**						
			**NaCl**	**KCl**	**LiCl**	**As(III)**	**As(V)**	**CdCl2**	**H2O2**
P. agglomerans PS1	5−9	10−50	1750mM	1750 mM	1750 mM	2000 PPM	1000 PPM	ND	10mM,
P.agglomerans PS2	4−9	10−50	2000mM	1750 mM	1750 mM	2000 PPM	1000 PPM	ND	12.5mM
P. agglomerans SP1	6.0−8.5	5−40	0.50%	ND	ND	100 mM	ND	ND	100µM
P.alhagi	5−9	7–48	9%	ND	ND	ND	ND	ND	ND
P. allii	4−9	30–40	ND	ND	ND	ND	ND	ND	ND
P. ananatis D1	4.8−7	20−40	100 mmol/L	ND	ND	ND	ND	ND	ND
P. anthophila BD 871	5−8	19 to 28	ND	ND	ND	ND	ND	ND	ND
P. sp. BS27	7−7.4	25−32	200mM	ND	ND	ND	ND	ND	ND
P. agglomerans lma2	4−8	30−44	300 mM	ND	50mM	ND	ND	ND	ND

Supplementary Table S4: Genomic assembly statistics of *Pantoea agglomerans* PS1 and PS2.

**Genome features**	***P. agglomerans* PS1**	***P. agglomerans* PS2**
Genome size	4987053 bp	5,177,646 bp
Contigs	96	406
CDS	4,860	5,275
GC	55.2	55.47
rRNA	9	11
tRNA	71	74
N50	428,608	562,669

Supplementary Table S5: Average nucleotide identity (ANI) of *Pantoea agglomerans* PS1 and PS2 with other *Pantoea* species. Here the Bac1 to Bac12 represents the different *Pantoea* species represented as Bac1: *agglomerans* FDAARGOS 1447, Bac 2: *agglomerans* AR1a, BAC 3: *CFSAN 047153*, Bac 4: *agglomerans* GB1, Bac 5: *agglomerans* Pa58, Bac 6: *rhizophila* JC1, Bac 7: rhizophila QL-P4, Bac 8: *pavanii* MHSD12, Bac 9: *pavanii* BWK1, Bac 10: *alhagi* LTYR11Z, Bac 11: ananatis LMG 20103, Bac 12: *ananatis* PA13, Bac 13: *ananatis* PA4, Bac 14: *brenneri* 11, Bac 15: *conspicua* LMG 24534, Bac 16: *deleyi* LMG 24200, Bac 17: *dispersa* Lsch, Bac 18: *eucalypti* NFPP29, Bac 19: *stewartii* DC283, Bac 20: *vagans* SRS89 151, Bac 21: *stewartii* M009.

	1	*P. agglomerans* PS1	*P.agglomerans* PS2	2	3	4	5	6	7	8	9	10	11	12	13	14	15	16	17
1	*	97.7 (92.68)	97.7 (92.72)	97.64 (92.63)	98.73 (93.77)	97.59 (65.57)	98.59 (94.31)	75.84 (63.19)	79.26 (75.94)	79.41 (74.65)	79.31 (75)	85.63 (80.66)	85.66 (84.6)	88.4 (82.68)	79.26 (72.95)	90.9 (87.07)	78.87 (62.26)	91.76 (88.33)	79.05 (74.25)
P.agglomerans PS1	96.52 (72.7)	*	98.64 (96.09)	98.44 (82.93)	96.51 (73.76)	95.91 (61.11)	96.36 (73.28)	75.21 (51.97)	78.34 (64.58)	78.65 (62.57)	78.39 (61.84)	84.46 (70.48)	84.57 (70.71)	86.93 (74.25)	78.49 (58.48)	89.81 (71.5)	77.9 (52.6)	90.51 (79.4)	78.25 (64.22)
P. agglomerans PS2	96.82 (75.21)	98.96 (89.35)	*	97.79 (75.39)	99.83 (76.2)	96.3 (63.11)	96.7 (75.82)	75.33 (53.33)	78.48 (66.37)	78.75 (64.37)	78.53 (63.54)	84.72 (72.76)	84.81 (72.71)	87.19 (76.42)	78.58 (60.28)	90.07 (73.86)	78.12 (54.01)	90.8 (82.05)	78.35 (65.86)
2	97.58 (88.74)	99.59 (99.09)	97.17 (89.05)	*	97.61 (88.59)	96.61 (61.26)	97.67 (89.3)	75.72 (61.2)	79.12 (72.98)	79.35 (71.94)	79.3 (72.56)	85.61 (77.93)	85.63 (81.06)	88.21 (79.75)	79.21 (69.98)	90.64 (84.28)	78.83 (60.45)	91.56 (84.98)	78.87 (71.57)
3	98.67 (92.41)	97.6 (92.56)	98.59 (92.57)	97.63 (91.04)	*	97.55 (65.33)	98.64 (92.17)	75.74 (62.7)	79.15 (74.63)	79.28 (73.37)	79.22 (73.81)	85.53 (80.37)	85.61 (83.7)	88.33 (82.49)	79.21 (72.15)	90.83 (86.49)	78.72 (61.96)	91.68 (87.34)	78.91 (73.58)

4	97.66 (74.17)	97.15 (88)	97.15 (87.97)	96.65 (72.62)	97.68 (74.89)	*	97.59 (74.57)	74.95 (50.89)	78.2 (64)	78.44 (60.44)	78.25 (60.96)	84.47 (75.44)	84.51 (70.53)	87.06 (74.45)	78.34 (57.29)	89.8 (72.02)	77.82 (53.65)	90.6 (81.08)	77.94 (62.49)
5	98.64 (92.24)	97.65 (91.43)	97.65 (91.46)	97.72 (91.41)	98.64 (92.12)	97.65 (64.12)	*	75.79 (62.05)	79.31 (74.74)	79.36 (73.7)	79.28 (74.03)	85.64 (80.02)	85.64 (83)	88.3 (82.01)	79.23 (71.62)	90.8 (86.02)	78.89 (62.07)	91.71 (87.76)	79.05 (72.89)
6	75.87 (59.82)	75.65 (62.72)	75.67 (62.76)	75.83 (59.91)	75.8 (60.17)	75.15 (42.85)	75.86 (59.6)	*	74.89 (59.82)	74.98 (58.24)	74.95 (58.35)	75.93 (58.13)	76.01 (59.67)	75.86 (60.02)	76.37 (58.85)	75.25 (58.91)	74.73 (50.49)	75.71 (61.06)	74.71 (59.03)
7	78.97 (65.16)	78.56 (70.4)	78.58 (70.41)	78.88 (65.25)	78.92 (65.39)	78.03 (48.79)	78.97 (65.6)	74.63 (54.53)	*	98.7 (88.99)	98.73 (89.21)	78.86 (63.8)	79.06 (64.51)	78.61 (66.76)	77.4 (60.92)	78.56 (65.49)	83.48 (68.15)	78.46 (68.68)	83.66 (83.23)
8	79.08 (65.99)	79.02 (69.75)	79.03 (69.97)	79.1 (66.01)	79.04 (66.02)	78.29 (47.43)	79.06 (66.41)	74.78 (54.17)	98.91 (90.92)	*	98.81 (91.53)	79.1 (63.46)	79.2 (65.57)	79.04 (67.6)	77.56 (61.23)	78.58 (66.06)	83.67 (66.79)	78.73 (67.38)	83.95 (81.87)
9	78.91 (61.88)	78.72 (64.63)	78.72 (64.66)	78.99 (62.26)	78.85 (62.1)	78.06 (44.3)	78.89 (62.17)	74.57 (50.73)	98.92 (85.73)	98.64 (86.6)	*	79.27 (60.16)	79.07 (61.27)	78.78 (62.69)	77.35 (57.09)	78.55 (62.68)	83.52 (63.26)	78.61 (63.21)	83.8 (77.54)
10	85.1 (69.57)	84.75 (77.55)	84.77 (77.56)	85.11 (70.38)	85.09 (70.55)	84.12 (57.41)	85.11 (70.54)	75.57 (52.87)	78.73 (64.17)	78.94 (62.13)	79.09 (63.24)	*	89.8 (76.92)	83.73 (70.56)	79.08 (60.22)	83.97 (70.17)	78.69 (53.37)	84.22 (74.65)	78.53 (63.07)
11	85.41 (78.32)	85.08 (83.94)	85.07 (83.99)	85.41 (78.91)	85.37 (79)	84.2 (57.88)	85.4 (78.78)	75.71 (58.62)	79.11 (70.11)	79.18 (69.12)	79.19 (68.92)	90.04 (82.95)	*	84.16 (76.04)	79.16 (67.2)	84.25 (78.01)	78.77 (59.36)	84.54 (81.3)	78.83 (68.79)
12	87.8 (73.91)	87.41 (83.97)	87.42 (83.96)	87.74 (74.05)	87.77 (74.78)	86.49 (59.18)	87.75 (74.42)	75.57 (55.98)	78.58 (69.06)	78.85 (67.91)	78.74 (67.27)	83.69 (72.83)	83.85 (73.27)	*	79.17 (62.57)	86.65 (75.67)	78.41 (57.51)	87.02 (81.49)	78.51 (69.43)

13	79.34 (72.13)	79.11 (73.88)	79.12 (73.91)	79.32 (72.59)	79.3 (72.65)	78.55 (50.58)	79.31 (72.25)	76.29 (61.64)	77.72 (70.48)	77.79 (68.91)	77.74 (69.01)	79.53 (69.21)	79.46 (71.82)	79.47 (70.22)	*	78.63 (72.19)	77.41 (58.23)	78.97 (72.08)	79.1 (69.18)
14	90.73 (84.9)	90.63 (88.31)	90.64 (88.31)	90.63 (85.41)	90.72 (85.61)	89.71 (61.63)	90.65 (85.3)	75.26 (60.83)	78.78 (74.63)	78.77 (73.33)	78.73 (73.4)	84.27 (78.67)	84.32 (81.35)	86.99 (82.31)	78.6 (70.83)	*	78.38 (61.83)	90.12 (85.75)	78.46 (73.09)
15	78.87 (57.93)	78.16 (62.9)	78.16 (62.92)	78.84 (58.5)	78.74 (58.63)	77.83 (44.27)	78.87 (58.39)	74.65 (49.24)	83.72 (74.87)	83.81 (71.6)	83.66 (73.63)	78.79 (58.07)	78.9 (59.46)	78.67 (63.49)	77.24 (54.89)	78.26 (60.04)	*	78.41 (61.67)	98.32 (82.47)
16	91.14 (77.45)	90.79 (88.85)	90.79 (88.83)	91.06 (77.58)	91.14 (77.59)	89.89 (63.02)	91.02 (78.38)	75.23 (56.69)	78.31 (69.92)	78.51 (67.05)	78.49 (67.33)	84.14 (75.6)	84.32 (76.33)	86.95 (80.2)	78.55 (63.42)	89.7 (77.39)	78.05 (58.04)	*	78.12 (69.11)
17	78.55 (62.32)	78.27 (68.47)	78.28 (68.5)	78.43 (62.38)	78.5 (63)	77.47 (47)	78.5 (62.44)	74.26 (52.28)	83.59 (81.19)	83.81 (78.09)	83.65 (78.2)	78.48 (61.21)	78.66 (61.82)	78.34 (65.91)	76.98 (57.98)	78.2 (63.17)	97.98 (71.4)	78.11 (66.06)	*

Supplementary Table S6: Tetra correlation among *Pantoea agglomerans* PS1 and PS2 and other *Pantoea* species by a wide distribution of Z-score.

Organism	Z-Score
Pantoea sp. CFSAN033090	0.99951
Pantoea pleuroti JZB 2120015	0.99946
Pantoea agglomerans 190	0.99936
Pantoea agglomerans GB1	0.99928
Pantoea agglomerans RIT273	0.99926
Pantoea agglomerans Eh318	0.99925
Pantoea agglomerans MP2	0.99921
Pantoea agglomerans Tx10	0.99919
Pantoea vagans ZBG6	0.99919
Pantoea agglomerans P10c	0.9991
Pantoea agglomerans FDAARGOS 1447	0.99903
Pantoea agglomerans NBRC 102470	0.99896
Pantoea vagans FDAARGOS_160	0.99856
Pantoea alfalfae CQ10	0.99843
Pantoea varia OV426	0.99837
*Pantoea vagan*s C9-1	0.99829
Pantoea vagans LMG 24199	0.99817
Pantoea gossypiicola B_8	0.99812
Pantoea vagans MP7	0.998
Pantoea eucalypti LMG 24197	0.99519
Pantoea eucalypti LMG 24197	0.99491
Pantoea sp. aB	0.99467
Pantoea agglomerans 299R	0.99438
Pantoea conspicua LMG 24534	0.98964
Pantoea anthophila LMG 2558	0.98806
Pantoea anthophila 11-2	0.98716
Pantoea sp. Sc1	0.98684
Pantoea brenneri LMG 5343	0.98577
Pantoea sp. 3.5.1	0.98525
Pantoea deleyi LMG 24200	0.97336
Pantoea deleyi LMG24200	0.97292
Pantoea deleyi LMG 24200	0.97266
[*Erwinia*] mediterraneensis Marseille-P5165	0.96327
Pantoea sp. PSNIH1	0.95662
Enterobacter cloacae subsp. cloacae ST436:951358951	0.95633
Erwinia sorbitola J780	0.95629
Lelliottia amnigena ZB04	0.95607
Enterobacter cloacae subsp. dissolvens GN02534	0.95558

Enterobacter cloacae subsp. cloacae SMART_855	0.95553
Enterobacter ludwigii Hanford	0.9553
Huaxiibacter chinensis 155047	0.95526
Pantoea eucrina LMG 5346	0.95515
Enterobacter ludwigii GN02226	0.95498
Enterobacter cloacae GGT036	0.95489
Enterobacter ludwigii GN02730	0.95483
Enterobacter cloacae subsp. cloacae GN02174	0.95463
Enterobacter sp. BIDMC 30	0.95462
Enterobacter ludwigii EcWSU1	0.9546
Enterobacter cloacae subsp. dissolvens ATCC 23373	0.9545
Enterobacter cloacae subsp. cloacae CIDEIMsCOL10	0.9545
Enterobacter cloacae subsp. cloacae SMART_1093	0.95441
Enterobacter ludwigii P101	0.95438
Enterobacter cloacae subsp. cloacae GN02616	0.95432
Enterobacter cloacae subsp. cloacae SMART_313	0.95426
Leclercia pneumoniae 49125	0.95411
Enterobacter cloacae subsp. cloacae SMART_901	0.95408
Enterobacter cloacae subsp. cloacae SMART_886	0.95404
Enterobacter ludwigii EN-119	0.95399
Enterobacter ludwigii UW5	0.95396
Enterobacter ludwigii GN04920	0.95391
Enterobacter cloacae B2	0.9539
Enterobacter cloacae subsp. cloacae SMART_1094	0.95384
Pantoea communis Al-1710	0.95375
Enterobacter cloacae subsp. cloacae 42324	0.95374
Enterobacter cloacae subsp. dissolvens SDM	0.95366
Enterobacter cloacae 6kgm	0.95355
Pseudocitrobacter faecalis DSM 27453	0.95351
*Enterobacte*r sp. 50793107	0.95345
Enterobacter cloacae ATCC 13047	0.95339
Enterobacter cloacae ATCC 13047	0.95321
Enterobacter cloacae subsp. cloacae ATCC 13047	0.95319
Enterobacter cloacae ATCC 13047	0.95309
Enterobacter cloacae DSM 30054	0.95309
Pantoea wallisii LMG 26277	0.95309
Enterobacter cloacae FDAARGOS 1431	0.95307
Enterobacter sp. BIDMC 26	0.95296
Enterobacter kobei DSM 13645	0.95273
Enterobacter cloacae CH1	0.95258
Duffyella gerundensis null	0.95222
Enterobacter kobei GN02266	0.9522
Enterobacter kobei SMART_635	0.95213
Enterobacteriaceae bacterium strain FGI 57	0.95208

*Enterobacter cancerogenu*s M004	0.95206
Enterobacter cloacae subsp. cloacae SMART_628	0.95197
Pseudocitrobacter corydidari	0.9519
Enterobacter kobei GN02275	0.95173
Enterobacter kobei GN03191	0.95172
Erwinia persicina NBRC 102418	0.95172
Enterobacter kobei MNCRE12	0.95167
Enterobacter kobei BIDMC 67	0.95166
*Enterobacter kobe*i e1326	0.95162
Enterobacter sp. MGH 25	0.95158
Enterobacter kobei GN02186	0.95151
Enterobacter kobei GN02225	0.9515
Enterobacter cloacae DSM 30054	0.9515
Enterobacter kobei GN02204	0.95149

Supplementary Table S7: Subsystem-related protein features for the resistance of metal/metalloid, antibiotics, and oxidative stress within the genome of *Pantoea agglomerans* PS1 and PS2.

**Nature of resistance**	**Identified protein feature in *P. agglomerans* PS1**	**Identified protein feature in *P. agglomerans* PS2**
Arsenic resistance	Arsenic resistance protein, ArsH, ACR3	Arsenic resistance protein ArsH
	Arsenic resistance operon repressor	Arsenical resistance operon repressor
	Arsenate reductase	Arsenate reductase
Cobalt-Zinc-Cadmium resistance	Cobalt-zinc-cadmium resistance protein CzcD	Cobalt-zinc-cadmium resistance protein CzcD
Hydroperoxide resistance	Organic hydroperoxide resistance protein	Organic hydroperoxide resistance protein
	Organic hydroperoxide resistance transcriptional regulator	Organic hydroperoxide resistance transcriptional regulator
Resistance to antibiotics	Multidrug efflux system MdtABC-TolC	Multidrug efflux system AcrAB-TolC

Supplementary Table S8: CAZymes encoding genes identified in *Pantoea agglomerans* PS1.

Sr no.	Enzyme family	Encoded enzymes	Hits
1	Auxiliary Activity Family 10	Lytic xylan monooxygenase / xylan oxidase (glycosidic bond-cleaving); Lytic chitin monooxygenase; Lytic cellulose monooxygenase (C1-hydroxylating); Lytic cellulose monooxygenase (C4-dehydrogenating)	2
2	Auxiliary Activity Family 1	[copper-containing] dihydrogeodin oxidase; Laccase / p-diphenol:oxygen oxidoreductase	6
3	Auxiliary Activity Family 3	Ecdysone oxidase (EC 1.1.3.16); Glucose 1-oxidase (EC 1.1.3.4); Aryl alcohol oxidase (EC 1.1.3.7); Oligosaccharide dehydrogenase (FAD) (EC 1.1.5.-); Glucose 1-dehydrogenase (FAD, quinone) (EC 1.1.5.9); Pyranose dehydrogenase (EC 1.1.99.29);	5
4	Auxiliary Activity Family 3 / Subf 2	Ecdysone oxidase;<br>Glucose 1-oxidase;<br>Aryl alcohol oxidase;<br>Oligosaccharide dehydrogenase (FAD);<br>Glucose 1-dehydrogenase (FAD, quinone);<br>Pyranose dehydrogenase;	3
5	Auxiliary Activity Family 6	P-benzoquinone reductase (NADPH);	2
6	Carbohydrate-Binding Module Family 48	Modules of approx. 100 residues with glycogen-binding function, appended to GH13 modules. Also found in the beta subunit (glycogen-binding) of AMP-activated protein kinases (AMPK)	9
7	Carbohydrate Esterase Family 1	Trehalose 6-O-mycolyltransferase;<br>Diacylglycerol O-acyltransferase;<br>Carboxylic ester hydrolase;<br>Acetylxylan esterase;<br>Feruloyl esterase	5
8	Carbohydrate Esterase Family 19	Pectin methylesterase	1
9	Carbohydrate Esterase Family 9	N-acetylglucosamine-6-phosphate deacetylase	3

10	Glycoside Hydrolase Family 1	anthocyanin 5-O-glucosyltransferase / 5-O-transglucosidase ; anthocyanin 7-O-glucosyltransferase / 7-O-transglucosidase ;β-transglucosidase;α-L-arabinopyranosidase ;β-1,2-glucosidase ;β-rutinosidase /α-L-rhamnose-(1,6)-β-D-glucosidase ;Isoflavone 7-O-glucosyl β-glucosidase;3-a-(S)-strictosidine β-glucosidase ;Lactase;Amygdalin β-glucosidase ;Prunasin β-glucosidase ; vicianin hydrolase ;Raucaffricine β-glucosidase;Myrosinase;βprimeverosidase;β-acuminosidase / β-apiosyl β-glucosidase / furcatin hydrolase ;ABA-specific β-glucosidase ;Exo-α-sialidase ;DIMBOA β-glucosidase ;Protodioscin 26-O-β-D-glucosidase ;0-26-avenacosidase ;Oleuropein β-glucosidase ;β-glucosidase;β-galactosidase;β-mannosidase;β-glucuronidase; Xylan β-1,4-xylosidase;β-D-fucosidase;β-glucosylceramidase;β-galactosylceramidase;Exo-β-1,3-glucanase;Exo-β-1,4-glucanase / cellodextrinase;6-P-β-galactosidase;6-P-β-glucosidase;	18
11	Glycoside Hydrolase Family 102	Peptidoglycan lytic transglycosylase / peptidoglycan lyase	3
12	Glycoside Hydrolase Family 103	Peptidoglycan lytic transglycosylase / peptidoglycan lyase	3
13	Glycoside Hydrolase Family 105	D-4,5-unsaturated α-galacturonidase;D-4,5-unsaturated β-glucuronyl hydrolase);Unsaturated rhamnogalacturonyl hydrolase	3
14	Glycoside Hydrolase Family 109	α-N-acetylgalactosaminidase;β-N-acetylhexosaminidase	3
15	Glycoside Hydrolase Family 13 / Subf 10	α-amylase; maltooligosyltrehalose trehalohydrolase	3
16	Glycoside Hydrolase Family 13 / Subf 18	α-glucoside phosphorylase; sucrose 6(F)-phosphate phosphorylase;Glucosylglycerate phosphorylase; glucosylglycerol phosphorylase;sucrose phosphorylase	2
17	Glycoside Hydrolase Family 13 / Subf 26	Malto-oligosyltrehalose synthase	2
18	Glycoside Hydrolase Family 13 / Subf 5	Maltopentaose-producing α-amylase ;] α-amylase; maltotriose-producing α-amylase; maltohexaose-producing α-amylase	3
19	Glycoside Hydrolase Family 15	Dextran dextrinase;[inverting] α-1,6-glucosidase / isomaltase;α,α-trehalase;[inverting] glucoamylase;Glucodextranase	9
20	Glycoside Hydrolase Family 177	Exo-α-sialidase	4
21	Glycoside Hydrolase Family 179	β-N-acetylhexosaminidase	3
22	Glycoside Hydrolase Family 19	Chitinase;Lysozyme;[reducing end] exo-chitinase	3

23	Glycoside Hydrolase Family 2	α-L-arabinopyranosidase;β-1,3-galactosidase;β-galacturonidase ;β-galacturonidase RGII specific;Lactase ; Glycyrrhizin β-glucuronidase ;β-D-galactofuranosidase ;Mannosylglycoprotein endo-β-mannosidase ;Exo-β-1,4-glucosaminidase ;β-glucosidase ;β-galactosidase ;β-mannosidase;β-glucuronidase ;Xylan β-1,4-xylosidase ;β-N-acetylhexosaminidase ;α-L-arabinofuranosidase	9
24	Glycoside Hydrolase Family 20	β-1,6-N-acetylglucosaminidase;β-N-acetyl-6-sulfo-glucosaminidase;Lacto-N-biosidase;β-N-acetylhexosaminidase;Mannosyl-glycoprotein endo-β-N-acetylglucosaminidase	6
25	Glycoside Hydrolase Family 23	Chitinase ;Lysozyme ;Peptidoglycan lytic transglycosylase / peptidoglycan lyase	20
26	Glycoside Hydrolase Family 24	Lysozyme	6
27	Glycoside Hydrolase Family 28	Xylogalacturonan hydrolase;Endo-polygalacturonase;Rhamnogalacturonase;Rhamnogalacturonan α-1,2-galacturonohydrolase;Galacturonan α-1,4-galacturonidase;Exo-poly-α-digalacturonosidase	3
28	Glycoside Hydrolase Family 3	β-NAcetylglucosaminoside phosphorylase;β-glycan phosphorylase;MLG exo-β-1,4-glucosidase;[retaining] xyloglucan-specific exo-β-1,4-glucosidase;α-L-arabinopyranosidase;Avenacinase;β-1,2-glucosidase;β-1,6-glucosidase;D-4,5-unsaturated β-glucuronyl hydrolase;Exo-β-1,3-1,4-glucanase;Stevioside-β-1,2-glucosidase;Tomatinase β-1,2-glucosidase;[retaining] isoprimeverose-producing oligoxyloglucan hydrolase;Coniferin β-glucosidase;Chitosanase;Protodioscin 26-O-β-D-glucosidase;β-glucosidase;β-galactosidase;β-glucuronidase;Xylan β-1,4-xylosidase;β-D-fucosidase;β-glucosylceramidase;β-N-acetylhexosaminidase;α-L-arabinofuranosidase;Exo-β-1,3-glucanase;Endo-β-1,3(4)-glucanase / lichenase-laminarinase;Lichenase /endo-β-1,3-1,4-glucanase;Exo-β-1,4-glucanase / cellodextrinase;Endo-β-1,4-xylanase	6
29	Glycoside Hydrolase Family 31 / Subf 2	Oligosaccharide α-4-glucosyltransferase;α-6-glucosyltransferase;α-3-isomaltosyltransferase / cycloalternan-forming enzyme;Exo-α-1,4-galactosidase;[retaining] oligo-α-1,6-glucosidase;Dextranase;α-xylosidase;Sulfoquinovosidase ;α-glucosidase;[retaining] cycloalternan-specific α-1,3-glucosidase / α-1,3-isomaltosidase ;Mannosyl-oligosaccharide α-1,3-glucosidase;Exo-acting protein-α-N-acetylgalactosaminidase ;α-galactosidase;α-mannosidase ;[retaining] sucrose α-glucosidase ;Exo-α-1,3-glucanase / α-1,3-glucosidase ;Exo-α-1,4-glucan lyase	3
30	GlycosylTransferase Family 84	Cyclic β-1,2-glucan synthase (elongation)	3

31	Glycoside Hydrolase Family 102	Peptidoglycan lytic transglycosylase / peptidoglycan lyase	3
32	GlycosylTransferase Family 20	GDP-valeniol: validamine 7-phosphate valeniolyltransferase;α,α-trehalose-6-phosphate synthase (UDP-forming);Glucosylglycerol-phosphate synthase;α,α-trehalose-6-phosphate synthase (GDP-forming);Trehalose-6-P phosphatase	3
33	GlycosylTransferase Family 35	α-1,4-glucan phosphorylase	3
34	Glycoside Hydrolase Family 77	4-α-glucanotransferase / amylomaltase	3
35	Glycoside Hydrolase Family 42	α-L-arabinopyranosidase;<br>β-galactosidase	3
36	Glycoside Hydrolase Family 53	Endo-β-1,4-galactanase	3
37	GlycosylTransferase Family 101	Hexosyltransferases	6
38	GlycosylTransferase Family 2	GDP-Man α-mannosyltransferase;GDP-Man: β-mannosyltransferase;GDP-β-L-Fuc : D-Glc-β-1,3-D-GalNAc-PP-Ph α-1,4-L-fucosyltransferase;Hexosyltransferases;LOS β-1,4-glucosyltransferase;N-acetylgalactosaminyltransferase;N-acetylglucosaminyltransferase;UDP-Gal: α-GlcNAc-PP-lipid β-1,3-galactosyltransferase;UDP-Gal:queuosine β-galactosyltransferase;UDP-GalNAc: β-1,3-N-acetylgalactosaminyltrasferase;UDP-GalNAc: undecaprenyl-P β-N-acetylgalactosaminyltransferase;UDP-Glc : GalNAc-PP-Und β-1,3-glucosyltransferase;UDP-Glc: β-1,6-glucan synthase;UDP-Glc: β-glucosyltransferase;UDP-Glc: bactoprenol β-glucosyltransferase;UDP-Glc: teichoic acid β-glucosyltransferase;UDP-GlcA: LOS β-glucuronyltransferase;UDP-GlcA: [hyaluronan] β-1,4-glucuronyltransferase;UDP-GlcNAc: [hyaluronan] β-1,3-N-acetylglucosaminyltransferase;[inverting] UDP-Glc: glycocin S-β-glucosyltransferase;[inverting] UDP-Glc: protein O-β-glucosyltransferas[inverting] UDP-GlcNAc: [chitooligosaccharide] β-1,4-N-acetylglucosaminyltransferase;[inverting] UDP-GlcNAc: teichoic acid β-N-acetylglucosaminyltransferase;[inverting] β-1,6-glucosyltransferase;[inverting] dTDP-spectinose: β-spectinosyltransferase;[inverting] glucomannan synthase;α-1,3-rhamnosyltransferase (unspecified donor);Alternating β-1,3/4-N-acetylmannan synthase;Arabinoglucan synthase;β-1,3-1,4-glucan synthase;β-1,3-N-acetylglucosaminyltransferase;β-1,3-galactosyltransferase;β-1,3-glucosyltransferase;β-1,4-galactosyltransferase;β-1,4-glucosyltransferase;Chlorobactene β-glucosyltransferase ;DTDP-L-Rha: α-L-rhamnosyltransferase;Poly-β-1,6-N-acetylglucosamine synthase ;Staphyloxanthin β-	9

	glucosyltransferase;Xyloglucan β-1,4-glucan synthase ;UDP-Glc: dolichyl-phosphate β-glucosyltransferase ;UDP-Glc: β-1,4-glucan synthase / cellulose synthase (UDP-forming) ;UDP-Glc: 1,2-diacylglycerol 3-glucosyltransferase ;Chitin transglycosylase ;UDP-GlcA: β-glucuronosyltransferase ;UDP-GalNAc: β-1,3-glucuronyl-N-acetylgalactosaminyl-proteoglycan β-1,4-N-acetylgalactosaminyltransferase ;β-Man-P-decaprenol: α-1,6-mannooligosaccharide α-1,6-mannosyltransferase;Hyaluronan synthase ;UDP-GlcA: N-acetylglucosaminyl-proteoglycan β-1,4-glucuronosyltransferase ;UDP-GlcA: N-acetylgalactosaminyl-proteoglycan β-1,3-glucuronosyltransferase ;UDP-Galf: rhamnopyranosyl-N-acetylglucosaminyl-PP-decaprenol β-1,4/1,5-galactofuranosyltransferase;UDP-Galf: [galactan] Galf-Galf-Rhα-GlcNAc-PP-decaprenol β-1,5/1,6-galactofuranosyltransferase ;DTDP-L-Rha: N-acetylglucosaminyl-PP-decaprenol α-1,3-L-rhamnosyltransferase ;β-1,3-glucosyltransferase;[inverting] GDP-Man: β-mannan β-1,4-mannosyltransferase / glucomannan synthase;Mannuronan synthase;UDP-Glc: β-1,3-glucan synthase / callose synthase ;GDP-Man: Dol-P β-mannosyltransferase;UDP-L-Arap-4N-formyl : undecaprenyl-P α-L-4N-formylarabinosyltransferase

Supplementary Table S9: CAZymes encoding genes identified in *Pantoea agglomerans* PS2.

Sr no.	Enzyme family	Encoded enzymes	Hits
1	Auxiliary Activity Family 1	dihydrogeodin oxidase;Laccase / p-diphenol:oxygen oxidoreductase	5
2	Auxiliary Activity Family 3	Pyranose:oxygen 2-oxidoreductase / glucose 2-oxidase ; Alcohol oxidase;Ecdysone oxidase ; Glucose 1-oxidase ; Aryl alcohol oxidase; Oligosaccharide dehydrogenase (FAD) ;Glucose 1-dehydrogenase (FAD, quinone);Cellobiose dehydrogenase ;Pyranose dehydrogenase	5
3	Auxiliary Activity Family 6	P-benzoquinone reductase	2
4	Carbohydrate-Binding Module Family 48	Modules of approx. 100 residues with glycogen-binding function, appended to GH13 modules. Also found in the beta subunit (glycogen-binding) of AMP-activated protein kinases (AMPK)	9
5	Carbohydrate Esterase Family 1	Trehalose 6-O-mycolyltransferase;Diacylglycerol O-acyltransferase ;Carboxylic ester hydrolase ;Acetylxylan esterase;Feruloyl esterase	2
6	Carbohydrate Esterase Family 11	UDP-3-O-acyl N-acetylglucosamine deacetylase	3
7	Carbohydrate Esterase Family 12	Carboxylic ester hydrolase ;Pectin acetylesterase ;Acetylxylan esterase ;Rhamnogalacturonan acetylesterase	2
8	Carbohydrate Esterase Family 19	Pectin methylesterase	4
9	Carbohydrate Esterase Family 4	Chitin oligosaccharide deacetylase;Acetylesterase ;Acetylxylan esterase ;LPS deacetylase ;Poly-β-1,6-N-acetylglucosamine deacetylase ;Peptidoglycan N-acetylglucosamine deacetylase ;Chitin deacetylase	2
10	Carbohydrate Esterase Family 9	N-acetylglucosamine-6-phosphate deacetylase	3

11	Glycoside Hydrolase Family 1	anthocyanin 5-O-glucosyltransferase / 5-O-transglucosidase ; anthocyanin 7-O-glucosyltransferase / 7-O-transglucosidase ;β-transglucosidase;α-L-arabinopyranosidase ;β-1,2-glucosidase ;β-rutinosidase /α-L-rhamnose-(1,6)-β-D-glucosidase ;Isoflavone 7-O-glucosyl β-glucosidase;3-a-(S)-strictosidine β-glucosidase ;Lactase;Amygdalin β-glucosidase ;Prunasin β-glucosidase ; vicianin hydrolase ;Raucaffricine β-glucosidase;Myrosinase;βprimeverosidase;β-acuminosidase / β-apiosyl β-glucosidase / furcatin hydrolase ;ABA-specific β-glucosidase ;Exo-α-sialidase ;DIMBOA β-glucosidase ;Protodioscin 26-O-β-D-glucosidase ;0-26-avenacosidase ;Oleuropein β-glucosidase ;β-glucosidase;β-galactosidase;β-mannosidase;β-glucuronidase; Xylan β-1,4-xylosidase;β-D-fucosidase;β-glucosylceramidase;β-galactosylceramidase;Exo-β-1,3-glucanase;Exo-β-1,4-glucanase / cellodextrinase;6-P-β-galactosidase;6-P-β-glucosidase;	18
12	Glycoside Hydrolase Family 102	Peptidoglycan lytic transglycosylase / peptidoglycan lyase	
13	Glycoside Hydrolase Family 103	Peptidoglycan lytic transglycosylase / peptidoglycan lyase	3
14	Glycoside Hydrolase Family 105	D-4,5-unsaturated α-galacturonidase ; D-4,5-unsaturated β-glucuronyl hydrolase ; Unsaturated rhamnogalacturonyl hydrolase	3
15	Glycoside Hydrolase Family 109	α-N-acetylgalactosaminidase ; β-N-acetylhexosaminidase	2
16	Glycoside Hydrolase Family 13 / Subf 10	[retaining] α-amylase; [retaining] maltooligosyltrehalose trehalohydrolase	3
17	Glycoside Hydrolase Family 13 / Subf 18	[retaining] α-glucoside phosphorylase ; [retaining] sucrose 6(F)-phosphate phosphorylase ;Glucosylglycerate phosphorylase ; [retaining] glucosylglycerol phosphorylase ; [retaining] sucrose phosphorylase	3
18	Glycoside Hydrolase Family 13 / Subf 26	Malto-oligosyltrehalose synthase	3

19	Glycoside Hydrolase Family 15	Dextran dextrinase ;[inverting] α-1,6-glucosidase / isomaltase ;α,α-trehalase;[inverting] glucoamylase;Glucodextranase	6
20	Glycoside Hydrolase Family 177	Exo-α-sialidase	7
21	Glycoside Hydrolase Family 179	β-N-acetylhexosaminidase	4
22	Glycoside Hydrolase Family 19	Chitinase;Lysozyme;[reducing end] exo-chitinase	3
23	Glycoside Hydrolase Family 2	α-L-arabinopyranosidase ;β-1,3-galactosidase ;β-galacturonidase ;β-galacturonidase RGII specific ;Lactase ;Glycyrrhizin β-glucuronidase; β-D-galactofuranosidase ;Mannosylglycoprotein endo-β-mannosidase;Exo-β-1,4-glucosaminidase;β-glucosidase;β-galactosidase;β-mannosidase;β-glucuronidase;Xylan β-1,4-xylosidase ;β-N-acetylhexosaminidase;α-L-arabinofuranosidase	6
24	Glycoside Hydrolase Family 20	β-1,6-N-acetylglucosaminidase ;β-N-acetyl-6-sulfo-glucosaminidase;Lacto-N-biosidase;β-N-acetylhexosaminidase ;Mannosyl-glycoprotein endo-β-N-acetylglucosaminidase	6
25	Glycoside Hydrolase Family 23	Chitinase ;Lysozyme ;Peptidoglycan lytic transglycosylase / peptidoglycan lyase	22
26	Glycoside Hydrolase Family 24	Lysozyme	6
27	Glycoside Hydrolase Family 28	Xylogalacturonan hydrolase (EC 3.2.1.-);Endo-polygalacturonase ;Rhamnogalacturonase ;Rhamnogalacturonan α-1,2-galacturonohydrolase;Galacturonan α-1,4-galacturonidase;Exo-poly-α-digalacturonosidase	2

28	Glycoside Hydrolase Family 3	β-NAcetylglucosaminoside phosphorylase;β-glycan phosphorylase;MLG exo-β-1,4-glucosidase;[retaining] xyloglucan-specific exo-β-1,4-glucosidase;α-L-arabinopyranosidase;Avenacinase;β-1,2-glucosidase;β-1,6-glucosidase;D-4,5-unsaturated β-glucuronyl hydrolase;Exo-β-1,3-1,4-glucanase;Stevioside-β-1,2-glucosidase;Tomatinase β-1,2-glucosidase;[retaining] isoprimeverose-producing oligoxyloglucan hydrolase;Coniferin β-glucosidase;Chitosanase;Protodioscin 26-O-β-D-glucosidase;β-glucosidase;β-galactosidase;β-glucuronidase;Xylan β-1,4-xylosidase;β-D-fucosidase;β-glucosylceramidase;β-N-acetylhexosaminidase;α-L-arabinofuranosidase;Exo-β-1,3-glucanase;Endo-β-1,3(4)-glucanase / lichenase-laminarinase;Lichenase /endo-β-1,3-1,4-glucanase;Exo-β-1,4-glucanase /cellodextrinase;Endo-β-1,4-xylanase	6
29	Glycoside Hydrolase Family 31 / Subf 2	Oligosaccharide α-4-glucosyltransferase;α-6-glucosyltransferase;α-3-isomaltosyltransferase / cycloalternan-forming enzyme;Exo-α-1,4-galactosidase;[retaining] oligo-α-1,6-glucosidase;Dextranase;α-xylosidase;Sulfoquinovosidase ;α-glucosidase;[retaining] cycloalternan-specific α-1,3-glucosidase / α-1,3-isomaltosidase ;Mannosyl-oligosaccharide α-1,3-glucosidase;Exo-acting protein-α-N-acetylgalactosaminidase ;α-galactosidase;α-mannosidase ;[retaining] sucrose α-glucosidase ;Exo-α-1,3-glucanase / α-1,3-glucosidase ;Exo-α-1,4-glucan lyase	3

Supplementary Table S10: Wheat Rhizospheric microbial diversity at different wheat growth stages.

	Feeks 1		
	**Control**	**PS1**	**PS2**
Acidobacteria	0.187059	0.670358	0.256772
Actinobacteria	2.295723	7.577966	4.326614
Aquificae	3.673157	0.058292	0.397997
Armatimonadetes	0	2.185952	0
Bacteroidetes	0.025508	0.058292	0.038516
Caldiserica	0	0.058292	0
Chlamydiae	0.144546	0.262314	0
Chlorobi	0.025508	0	0.077032
Chloroflexi	1.088343	0.29146	0.051354
Chordata	0.051016	0.058292	0
Chrysiogenetes	0.017005	0	0
Cyanobacteria	0.161551	0.466336	0.064193
Deferribacteres	0.025508	0	0
Deinococcus-Thermus	0.153048	0.058292	0.564899
Elusimicrobia	0	0.058292	0
Euryarchaeota	34.58039	0	13.33932
Fibrobacteres	10.23722	0.174876	14.76441
Firmicutes	5.824335	20.69368	9.872898
Fusobacteria	0.093529	0.087438	0
Gemmatimonadetes	0	0.728651	0.051354
Nitrospirae	0.017005	0	0
Planctomycetes	0.042513	0.058292	0
Proteobacteria	41.20398	62.83882	56.1176
Spirochaetes	0.034011	0.29146	0.025677
Synergistetes	0	0.14573	0
Tenericutes	0.059519	0.408044	0.025677
Thermodesulfobacteria	0.017005	0.058292	0
Thermotogae	0.017005	2.593996	0.025677
Verrucomicrobia	0.025508	0.116584	0
	**Feeks 2**		
	**Control**	**PS1**	**PS2**
Acidobacteria	0.466931	1.710558	1.946794
Actinobacteria	5.916592	6.098091	15.02
Aquificae	0.179097	0.198115	0.155933
Armatimonadetes	0.019189	0.072481	0.085054
Bacteroidetes	2.136369	10.39865	2.256
Caldiserica	0.031982	0.019328	0.014176
Candidatus Cloacimonetes	0.019189	0.014496	0.023626
Chlamydiae	0.076756	0.067649	0.075604
Chlorobi	0.134323	0.14013	0.174833
Chloroflexi	1.586286	3.020053	3.369088

Chordata	0.921069	1.299831	1.152956
Chrysiogenetes	0.051171	0.028993	0.028351
Crenarchaeota	0	0.009664	0.018901
Cyanobacteria	1.918895	1.560763	
Deferribacteres	0.025585	0.077313	0.141757
Deinococcus-Thermus	0.300627	0.488041	0.283514
Dictyoglomi	0.031982		0.00945
Elusimicrobia	0.044774		0.151207
Euryarchaeota	0.057567	0.038657	0.066153
Fibrobacteres	0.044774	0.02416	0.051978
Firmicutes	12.63272	11.95458	8.977933
Fusobacteria	0.089548	0.09181	0.184284
Gemmatimonadetes	0.204682	0.531529	0.637906
Ignavibacteriae	5.2		0.051978
Nitrospirae	3		
Planctomycetes	0.575668	0.444552	1.691632
Proteobacteria	56.90162	60.72	62.6403
Spirochaetes	0.5422	0.647499	0.533951
Synergistetes	0.095945		
Tenericutes	0.217475	0.329	
Thaumarchaeota	0	0.009664	0
Thermodesulfobacteria	2	0.005	0.051978
Thermotogae	3		0.2047
Verrucomicrobia	1.577		0

Feeks 3

	Control	PS1	PS2
Acidobacteria	2.668737	2.737992	1.563308
Actinobacteria	15.07448	11.85192	9.350285
Aquificae	0.347385	0.237954	0.646759
Armatimonadetes	0.431167	0.207447	0.048045
Bacteroidetes	8.811329	12.67103	7.942198
Caldiserica	0.034739	0.028982	0.02587
Candidatus Cloacimonetes	0.030652	0.04576	0.044349
Chlamydiae	0.11852	0.070166	0.062828
Chlorobi	0.259517	0.17694	0.214354
Chloroflexi	3.843717	2.890526	4.253825
Chordata	0.132824	0.155585	0.291965
Chrysiogenetes	0.055173	0.050336	0.036958
Crenarchaeota	0.016348	0.018304	0.033262
Cyanobacteria	2.513436	1.722113	2.80139
Deferribacteres	0.159389	0.149484	0.162614
Deinococcus-Thermus	0.584425	0.462179	0.417621
Dictyoglomi	0.055173	0.033558	0.036958
Elusimicrobia	0.273821	0.196769	0.181092
Euryarchaeota	0.040869	0.039659	0.044349
Fibrobacteres	0.081738	0.050336	0.029566
Firmicutes	8.533421	6.769475	8.962229
Fusobacteria	0.167562	0.106774	0.262399
Gemmatimonadetes	1.101416	0.774875	0.535886

Ignavibacteriae	0.136911	0.073216	0.055436
Nitrospirae	0.782639	0.584207	0.554365
Planctomycetes	2.023009	1.528394	2.165718
Proteobacteria	46.98899	51.54594	55.02624
Spirochaetes	0.821464	0.719962	0.813068
Synergistetes	0.300386	0.186092	0.203267
Tenericutes	0.398472	0.215073	0.546973
Thermodesulfobacteria	0.128737	0.11135	0.151526
Thermotogae	0.437297	0.279138	0.251312
Verrucomicrobia	2.646259	3.30847	2.283983
**Feeks 6**			
	**Control**	**PS1**	**PS2**
Acidobacteria	1.268961	1.617436	1.716751
Actinobacteria	3.563175	10.33266	2.920589
Aquificae	0.239506	0.174935	0.162925
Armatimonadetes	0.121854	0.197878	0.099566
Bacteroidetes	13.71906	18.95325	15.23051
Caldiserica	0.025211	0.028678	0.009051
Candidatus Cloacimonetes	0.033615	0.020075	0.012069
Chlamydiae	0.092441	0.100373	0.066377
Chlorobi	0.176478	0.11758	0.141805
Chloroflexi	2.374049	1.866934	1.583997
Chordata	0.277323	0.180671	0.171977
Chrysiogenetes	0.042019	0.020075	0.033189
Crenarchaeota	0.012606	0	0.012069
Cyanobacteria	4.067398	1.617436	1.004707
Deferribacteres	0.121854	0.057356	0.051291
Deinococcus-Thermus	0.52103	0.324061	0.280594
Dictyoglomi	0.008404	0.031546	0.018103
Elusimicrobia	0.168074	0.206481	0.114651
Euryarchaeota	0.050422	0.022942	0.036206
Fibrobacteres	5	0.060224	0.039223
Firmicutes	7.811253	5.334098	6.495897
Fusobacteria	0.201689	0.108976	0.075428
Gemmatimonadetes	0.369763	0.455979	0.265508
Ignavibacteriae	0.189084	0.120447	0.096548
Nitrospirae	0.537838	0.679667	0.398262
Planctomycetes	2.151351	0.748494	0.524982
Proteobacteria	47	53.75108	66.86278
Spirochaetes	0.920207	0.576427	0.509896
Synergistetes	0.210093	0.131919	0.102583
Tenericutes	2.59	0.31259	0.181028
Thermodesulfobacteria	0.11345	0.068827	
Thermotogae	4	0.252366	0.153874
Verrucomicrobia	2.029497	1.528535	0.63
**Feeks 9**			
	**Control**	**PS1**	**PS2**
Acidobacteria	2.37521	3.070208	2.678325
Actinobacteria	5.83578	5.643766	6.044374
Aquificae	0.524329	0.221988	0.28684

Armatimonadetes	0.534815	0.10535	0.165883
Bacteroidetes	8.410235	13.73316	16.7473
Caldiserica	0.041946	0.033863	0.041471
Candidatus Cloacimonetes	0.036703	0.01505	0.034559
Chlamydiae	0.146812	0.082775	0.065662
Chlorobi	0.33557	0.142975	0.235001
Chloroflexi	4.205117	2.57732	3.006635
Chordata	0.346057	0.1204	0.124412
Chrysiogenetes	0.089136	0.033863	0.082942
Crenarchaeota	0.026216	0.033863	0.027647
Cyanobacteria	1.316065	2.71277	0.915814
Deferribacteres	0.183515	0.109113	0.15206
Deinococcus-Thermus	0.681628	0.342388	0.525297
Dictyoglomi	0.036703	0.04515	0.034559
Elusimicrobia	0.597735	0.124163	0.214266
Euryarchaeota	0.094379	0.04515	0.048383
Fibrobacteres	0.104866	0.033863	0.079486
Firmicutes	10.17722	8.853187	7.461294
Fusobacteria	5.23	0.139213	0.093309
Gemmatimonadetes	1.431418	0.301001	1.14736
Ignavibacteriae	0.277894	0.097825	0.117501
Nitrospirae	1.856124	0.481601	0.829417
Planctomycetes	3.880034	1.659267	0.76721
Proteobacteria	42.693	57.55136	55.99599
Spirochaetes	0.996225	0.628339	0.642798
Synergistetes	0.461409	0.158025	0.138236
Tenericutes	2	0.331101	0.314487
Thaumarchaeota	2.51	0	0.010368
Thermodesulfobacteria	0.262164	0.071488	0.127868
Thermotogae	0.786493	0.237038	0.0653
Verrucomicrobia	1.516	0.27	0.78
**Feeks 10.5**			
	**Control**	**PS1**	**PS2**
Acidobacteria	1.673319	2.437223	2.263856
Actinobacteria	5.802886	8.788774	7.272964
Aquificae	0.583359	0.196947	0.520427
Armatimonadetes	0.49125	0.410307	0.4879
Bacteroidetes	5.403746	10.43821	7.786885
Caldiserica	0.092109	0.016412	0.039032
Candidatus Cloacimonetes	0.015352	0.057443	0.058548
Chlamydiae	0.230273	0.049237	0.169139
Chlorobi	0.506601	0.205153	0.422847
Chloroflexi	5.34234	5.0796	5.035129
Chordata	0.445195	0.155917	0.227687
Chrysiogenetes	0.076758	0.065649	0.058548
Crenarchaeota	5.36	0.016412	0.065053

Cyanobacteria	4.35984	2.420811	1.444184
Deferribacteres	5.26	0.10668	0.279729
Deinococcus-Thermus	1.197421	0.311833	1.014832
Dictyoglomi	0.184219	0.041031	0.071559
Elusimicrobia	0.629414	0.21336	0.637523
Euryarchaeota	5.099	0.082061	0.084569
Fibrobacteres	0.076758	0.049237	0.123601
Firmicutes	7.706478	8.772362	8.26828
Fusobacteria	0.260976	0.057443	0.227687
Gemmatimonadetes	1.120663	0.82882	1.535259
Ignavibacteriae	0.337734	0.155917	0.279729
Nitrospirae	2.195272	1.329394	2.992454
Planctomycetes	1.136015	1.04218	1.762946
Proteobacteria	40	53.95536	53.35675
Spirochaetes	0.337734	0.541605	0.565964
Synergistetes	0.39914	0.180535	0.435857
Tenericutes	1.128	0.61546	0.513921
Thermodesulfobacteria	0.138164	0.073855	0.065053
Thermotogae	0.644765	0.328246	0.715587
Verrucomicrobia	1.765428	0.97653	1.216498

Supplementary Figure SF1: Growth pattern analysis of *Pantoea agglomerans* PS1 (A) *and* PS2 (B) after incubating the cultures for 48 hours in LB broth with constant shaking at 200 rpm. Experiments were carried out in triplicates and growth was observed by taking absorbance at 600nm after an interval of 4 hours. Plotted values are the mean of triplicates along with the observed standard deviation.

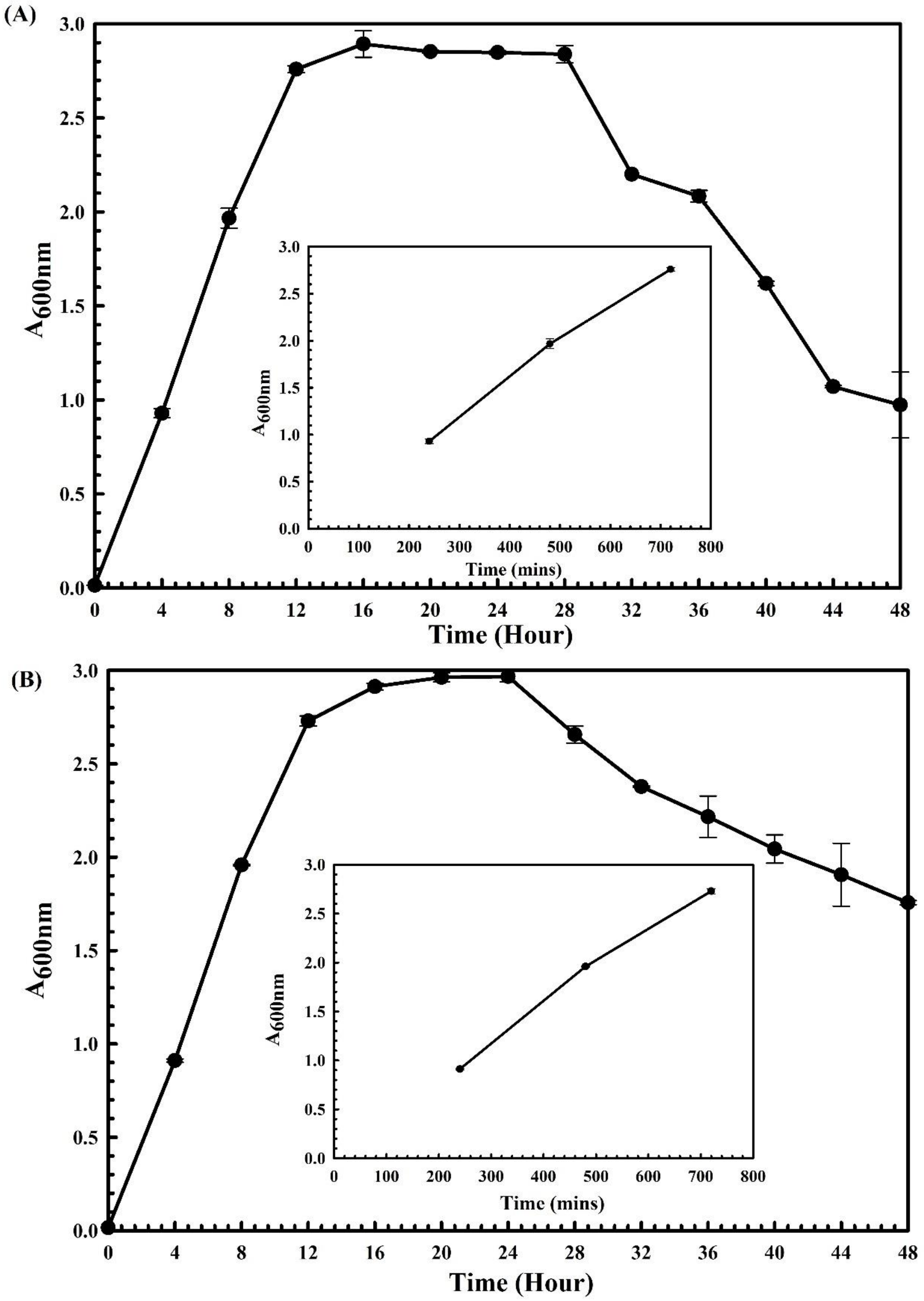

Supplementary Figure SF2: Total rhizospheric sugar at different wheat growth stages. Total sugar content estimated with the anthrone method at different feeks (1.0, 2.0, 3.0, 6.0, 9.0, 10.5) in the presence and the absence of microbial inoculants PS1(A) and PS2 (B), respectively. Reducing sugar content (estimated using DNS assay) at different feeks (1.0, 2.0, 3.0, 6.0, 9.0, 10.5) in the presence and the absence of microbial inoculants PS1 (C) and PS2 (D), respectively (**B**). Here control is specific to each feek for appropriate data presentation.. Experiments were carried out in triplicates and read at 620nm. Plotted values are the mean of triplicate readings along with their observed standard deviation.

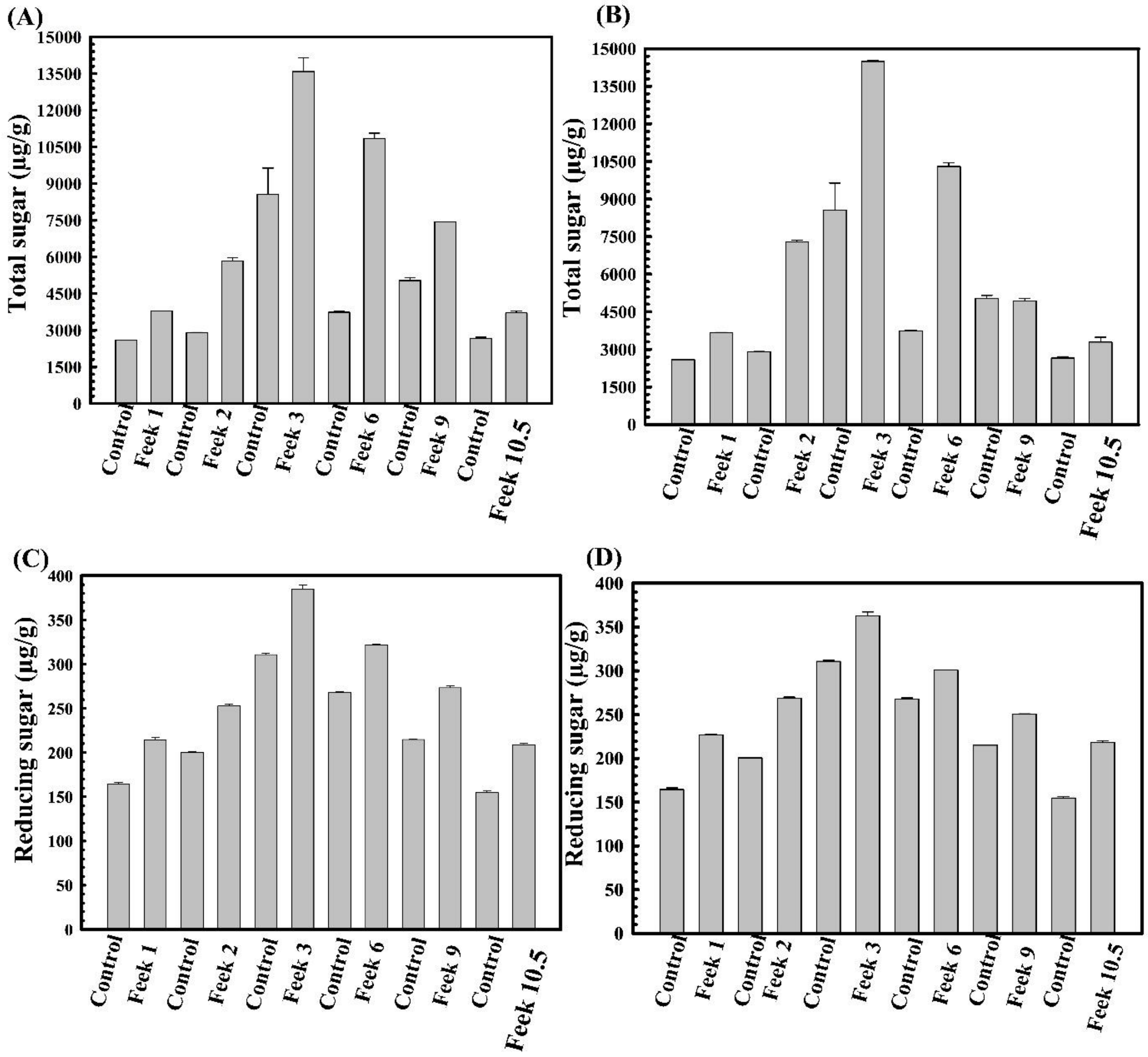

Supplement: Supplementary file 1 [file Data_Sheet_1.PDF]
